# Identification of a novel GR-ARID1a-P53BP1 protein complex involved in DNA damage repair and cell cycle regulation

**DOI:** 10.1038/s41388-022-02516-2

**Published:** 2022-11-07

**Authors:** Felicity E. Stubbs, Benjamin P. Flynn, Caroline A. Rivers, Matthew T. Birnie, Andrew Herman, Erin E. Swinstead, Songjoon Baek, Hai Fang, Jillian Temple, Jason S. Carroll, Gordon L. Hager, Stafford L. Lightman, Becky L. Conway-Campbell

**Affiliations:** 1 Henry Wellcome Laboratories for Integrative Neuroscience and Endocrinology, Translational Health Sciences, Faculty of Health Sciences, University of Bristol, Dorothy Hodgkin Building, Whitson Street, Bristol, BS1 3NY UK; 2 Laboratory of Receptor Biology and Gene Expression, The National Cancer Institute, US National Institutes of Health, 41 Medlars Drive, Bethesda, MD 20892 USA; 3 Flow Cytometry Facility, Faculty of Life Sciences, School of Cellular & Molecular Medicine, Biomedical Sciences Building, University of Bristol, Bristol, BS8 1TD UK; 4 Shanghai Institute of Hematology, State Key Laboratory of Medical Genomics, National Research Center for Translational Medicine at Shanghai, Ruijin Hospital, Shanghai Jiao Tong University School of Medicine, Shanghai, 200025 China; 5 Cancer Research UK Cambridge Institute, University of Cambridge, Robinson Way, Cambridge, CB2 0RE UK

**Keywords:** Oncogenes, Cell growth, Predictive markers

## Abstract

ARID1a (BAF250), a component of human SWI/SNF chromatin remodeling complexes, is frequently mutated across numerous cancers, and its loss of function has been putatively linked to glucocorticoid resistance. Here, we interrogate the impact of siRNA knockdown of ARID1a compared to a functional interference approach in the HeLa human cervical cancer cell line. We report that ARID1a knockdown resulted in a significant global decrease in chromatin accessibility in ATAC-Seq analysis, as well as affecting a subset of genome-wide GR binding sites determined by analyzing GR ChIP-Seq data. Interestingly, the specific effects on gene expression were limited to a relatively small subset of glucocorticoid-regulated genes, notably those involved in cell cycle regulation and DNA repair. The vast majority of glucocorticoid-regulated genes were largely unaffected by ARID1a knockdown or functional interference, consistent with a more specific role for ARID1a in glucocorticoid function than previously speculated. Using liquid chromatography-mass spectrometry, we have identified a chromatin-associated protein complex comprising GR, ARID1a, and several DNA damage repair proteins including P53 binding protein 1 (P53BP1), Poly(ADP-Ribose) Polymerase 1 (PARP1), DNA damage-binding protein 1 (DDB1), DNA mismatch repair protein MSH6 and splicing factor proline and glutamine-rich protein (SFPQ), as well as the histone acetyltransferase KAT7, an epigenetic regulator of steroid-dependent transcription, DNA damage repair and cell cycle regulation. Not only was this protein complex ablated with both ARID1a knockdown and functional interference, but spontaneously arising DNA damage was also found to accumulate in a manner consistent with impaired DNA damage repair mechanisms. Recovery from dexamethasone-dependent cell cycle arrest was also significantly impaired. Taken together, our data demonstrate that although glucocorticoids can still promote cell cycle arrest in the absence of ARID1a, the purpose of this arrest to allow time for DNA damage repair is hindered.

## Introduction

Glucocorticoids are widely used in the clinic due to their potent anti-inflammatory properties and are extensively used as a cancer treatment due to their ability to induce apoptosis and promote cell cycle arrest [1–3]. However, the benefits of glucocorticoids must be balanced against the many adverse side effects, as well as the development of glucocorticoid resistance in some patients. Mutations in a BRG1 associated factor (BAF, also known as the human SWItch/Sucrose Non-Fermentable (hSWI/SNF)) chromatin remodeling complex subunit AT-Rich Interaction Domain 1A (ARID1a/BAF250) have been associated with glucocorticoid resistance [4]; however, the exact role of ARID1a in GR signaling is unknown. Mutations in ARID1a have been identified in multiple human carcinomas [5–9] at an alarmingly high incidence, and recently compared to the mutation frequency of the P53 gene (TP53) which is the most commonly altered gene in human carcinomas [10, 11]. Notably, the C-terminal of ARID1a has been shown to directly bind the glucocorticoid receptor (GR) [12, 13] (Supplementary Fig. S1a) as well as to P53 [5] and DNA repair protein kinase ATR [14] but there has been a lack of evidence to determine a role of ARID1a in GR-mediated cell cycle arrest and DNA repair.

Chromatin remodeling by the BAF (hSWI/SNF) complex is important for genomic GR signaling, with chromatin accessibility being dynamically altered at GR binding sites in target genes to regulate transcription [15]. The Mouse Mammary Tumor Virus (MMTV) array was initially used to assess the actions of the SWI/SNF complex on GR transcriptional activity [16, 17]. At the MMTV array, GR binds to glucocorticoid response elements (GREs) and recruits the SWI/SNF complex to the chromatin to promote nucleosome reorganization and increase access for RNA Polymerase II and associated transcriptional machinery [18]. ARID1a has been suggested to mediate this role and confer specificity to the SWI/SNF complex for GR-dependent gene regulation [12, 13]. Additionally, the SWI/SNF complex is essential in pre-establishing chromatin accessibility, which largely determines GR binding [15, 19]. Transcription factors such as C/EBPβ, AP1, and FOXA1 also pre-occupy the chromatin around binding sites for GR and other nuclear receptors; in a similar way, ARID1a may establish and maintain cell-specific chromatin accessibility [19–22]. Previous studies using reporter assays have shown that removal of the C-terminus of ARID1a decreases GR activity [12, 13]. Interestingly, overexpression of the ARID1a C-terminal domain (ARID1a-CTD) also impairs GR activity [12, 13]; indicating a requirement for both N- and C-terminal regions of ARID1a for full GR activity. The ARID1a-CTD is essential for binding the BAF complex core ATPase, BRG1 [12, 23], and therefore will be incorporated into the complex despite loss of the N-terminal domain preventing binding of the full length ARID1a. Thus, overexpression of this GR-interacting ARID1a C-terminal domain acts in a dominant negative manner, functionally interfering with ARID1a.

Recently we found that the SKOV3 ovarian cancer cell line, which harbors a truncating ARID1a mutation, exhibited a highly restricted glucocorticoid transcriptional response [24]. We therefore tested whether ARID1a siRNA knockdown, or functional interference by overexpression of the GR-interacting C-terminal domain of ARID1a (Supplementary Fig. S1d,e) impairs regulation of GR-dependent genes in the otherwise robustly glucocorticoid-responsive HeLa cell line. We report that ARID1a knockdown caused significant genome-wide alterations in GR binding profiles and chromatin accessibility, impacting upon a specific subset of GR target genes important for glucocorticoid regulation of cell cycle and DNA repair; the functional consequence of which was primarily related to an increase in DNA damage and prolonged cell cycle arrest. Finally, we demonstrate that ARID1a is required for the formation of a macromolecular complex consisting of GR, P53BP1, DNA repair proteins including Poly(ADP-Ribose) Polymerase 1 (PARP1), DNA damage-binding protein 1 (DDB1), DNA mismatch repair protein MSH6 and splicing factor proline and glutamine-rich protein (SFPQ). Also found in the complex, histone acetyltransferase KAT7 is an epigenetic regulator of steroid-dependent transcription, DNA damage repair and P53-dependent cell cycle regulation. This is the first evidence for a role of ARID1a in GR-mediated DNA repair. Taken together, our findings provide new insights into how loss of ARID1a impairs glucocorticoid-mediated DNA damage repair and prolongs glucocorticoid-dependent cell cycle arrest in response to increasing DNA damage.

## Results

### Significant changes in genome-wide GR binding following ARID1a knockdown

**Fig. 1:**
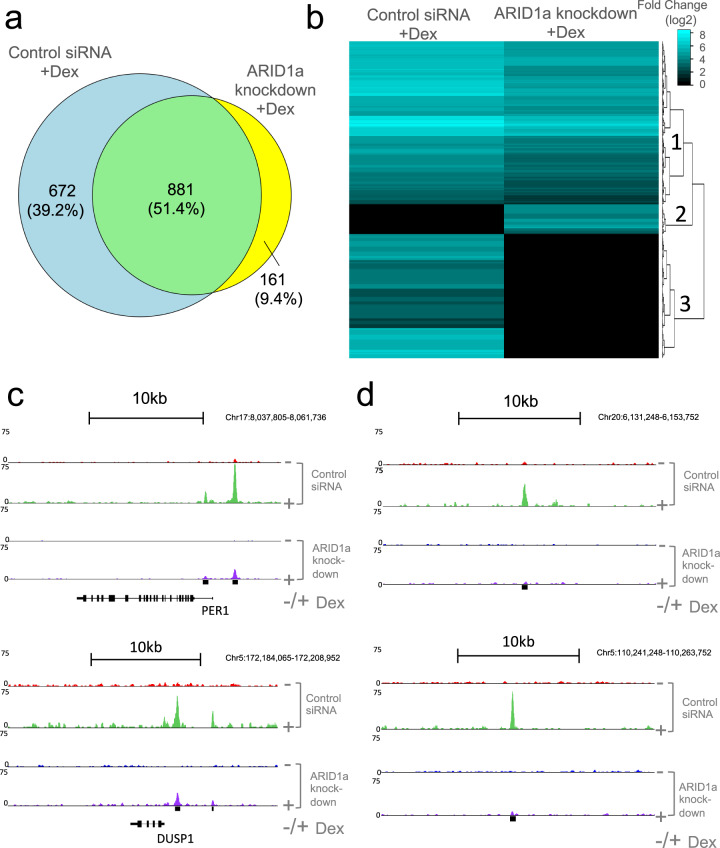
Significant decrease in GR binding in the presence of ARID1a siRNA. **a** Venn diagram and **b** hierarchically clustered heatmap of all significant (*p* < 0.05) Dex-induced GR binding events (30 min 100 nM Dex treatment) in control siRNA or ARID1a siRNA knockdown cells. The main clusters identified: Cluster 1) Significant Dex-induced GR binding in both control siRNA and ARID1a knockdown groups; Cluster 2) Significant Dex-induced GR binding only in ARID1a knockdown group); Cluster 3) Significant Dex-induced GR binding detected in control siRNA only. GR enrichment calculated by Log2 fold change relative to respective groups without Dex treatment (>0.585 Log2 fold change and adjusted *p* < 0.05). **c** Genome browser tracks show representative examples of cluster 1 GR binding sites at PER1 and DUSP1 loci. **d** Genome browser tracks show representative examples of cluster 3 GR binding sites.

To assess the impact of ARID1a knockdown on dexamethasone (Dex)-induced GR binding, chromatin samples (nine samples each comprising 100 μg chromatin) were immunoprecipitated with GR antibody, and the resulting immunoprecipitates were combined for library preparation and sequencing. Two separate ChIP-Seq experiments were performed independently to allow for concordant peak calling analysis as previously described [25]; the GR ChIP-Seq data were first normalized to input chromatin then sites of enrichment were identified using findPeaks (HOMER v4.9.1). This analysis detected 1714 GR binding sites in total, of which 881 (51.4% of all identified sites) retained significant GR binding after ARID1a knockdown. The remaining 833 identified GR binding sites were significantly impacted by ARID1a knockdown; GR binding was lost at 672 sites (39.2% of all identified sites) while a gain in GR binding was found at 161 sites (9.4% of total) (Fig. 1a).

The data are visualized in the hierarchical clustering heatmap (Fig. 1b). Cluster 1 contains sites where significant GR binding was detected in both control and ARID1a knockdohwn conditions (common GR binding sites). Cluster 2 (gained GR binding) contains loci where increased GR binding was detected with ARID1a knockdown, and cluster 3 (lost GR binding) contains sites where a significant decrease in GR binding was detected with ARID1a knockdown. Supplementary Fig. S2a–c shows further interrogation of these data, which indicated that differential GR binding intensity was strongest in cluster 3. Typical examples of common GR binding sites (cluster 1) are shown in Fig. 1c, where significant GR binding remained detectable at known proximal GREs in highly inducible glucocorticoid-regulated genes including PER1 and DUSP1 (Fig. 1c). Examples of ARID1a-dependent differential GR binding sites (cluster 3) are shown in Fig. 1d. The sites shown are at intragenic sites without proximity to annotated genes. Finally, no differential GR binding was detected between ARID1a knockdown and siRNA control in the absence of Dex (Supplementary Fig. S2d).

### Significant changes in chromatin accessibility with ARID1a knockdown

**Fig. 2:**
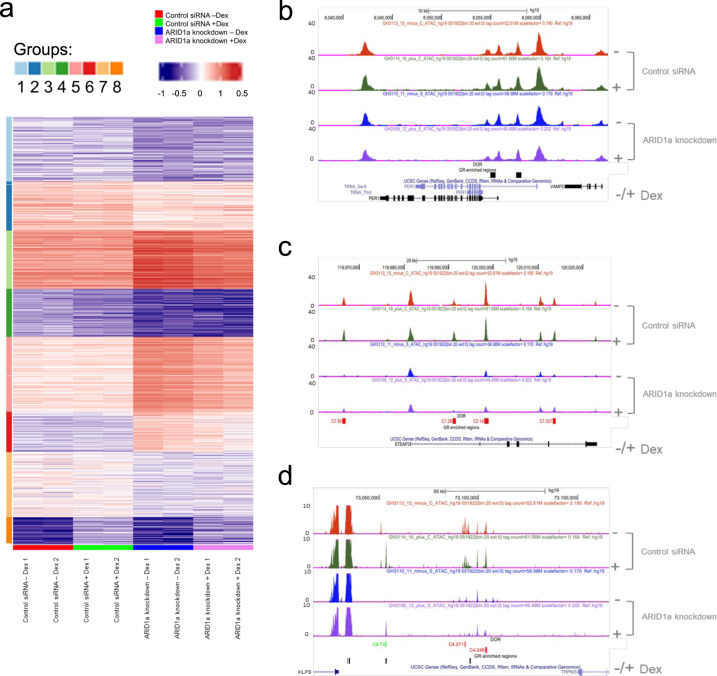
Effects on chromatin accessibility following ARID1a knockdown. **a** Heatmap showing Differentially Open Regions (DORs) on a global scale. Significant DORs were detected using DESeq algorithm, then clustered by K-means algorithm. Samples are clustered by a hierarchical clustering algorithm using the Euclidean distance measure. 8 clusters are observed; clusters 1, 2, 4, and 7 show reduced pre-accessibility in ARID1a knockdown cells, clusters 3, 5, and 6 show increased pre-accessibility in ARID1a knockdown cells, cluster 8 includes Dex-inducible sites in both control and ARID1a knockdown cells. **b** ATAC-Seq track for PER1, an example of a Dex-inducible gene, with location of GR binding sites shown on lower track. Multiple pre-accessible sites are evident, but no DORs were detected in this locus. **c** ATAC-Seq track for STEAP3 with DORs (representing clusters 1, 2, and 7) indicated. There are no GR binding sites in this region. **d** ATAC-Seq track of KLF9 with DORs (representing cluster 8 and cluster 4) shown, and location of GR binding sites indicated.

As GR binding influences subsequent chromatin accessibility, we hypothesized that the observed ARID1a-dependent global changes in GR binding would also impact upon global chromatin accessibility. We therefore performed ATAC-Seq, with two independent replicates for each of the four conditions (-Dex baseline and +Dex GR induction for each of ARID1a knockdown and control siRNA conditions), which revealed genome-wide changes in chromatin accessibility upon knockdown of ARID1a (Fig. 2).

K-means clustering of differentially open regions (DORs) identified 8 discrete cluster groups, each of which describes specific combinations of pre-accessibility and Dex-inducibility for each of the four conditions (Z-score heatmap is shown in Fig. 2a and Tag density heatmap is shown in Supplementary Fig. S3a). The largest clusters (group1, 811 sites; group2, 515 sites; group4 516 sites; group7, 691 sites) revealed a generalized decrease in chromatin pre-accessibility in ARID1a knockdown cells. There were also clusters with increased pre-accessibility in ARID1a knockdown cells (group3, 530 sites; group5, 826 sites; group6, 393 sites). Finally, there was a small but notable group of Dex-inducible sites, which were found in both control and ARID1a knockdown (Group 8, 285 sites). Taken together, these data suggest that ARID1a plays a greater role in establishing chromatin pre-accessibility than in Dex-induced chromatin remodeling.

Genome browser shots of ATAC-Seq data show typical examples of loci from some of the notable clusters, with annotated tracks indicating location of differentially open regions (DORs) and GR binding site locations from the GR ChIP-Seq analysis. The PER1 gene locus (Fig. 2b) contains pre-accessible sites but no DORs, the STEAP3 gene locus (Fig. 2c) contains annotated DORs with decreased pre-accessibility in ARID1a knockdown cells (groups 1, 2, and 7), and the KLF9 gene locus (Fig. 2d) contains two annotated DORs with decreased pre-accessibility in ARID1a knockdown cells (group 4) and one Dex-inducible annotated DOR (group 8) found in both ARID1a knockdown and control cells. Supplementary Fig. 3b shows motif analysis which indicates that the Dex-inducible sites (cluster iii; group 8) are more highly enriched in GRE motifs than the other groups (clusters i and ii; groups 1–7).

### Limited role for ARID1a in glucocorticoid-target gene regulation

**Fig. 3:**
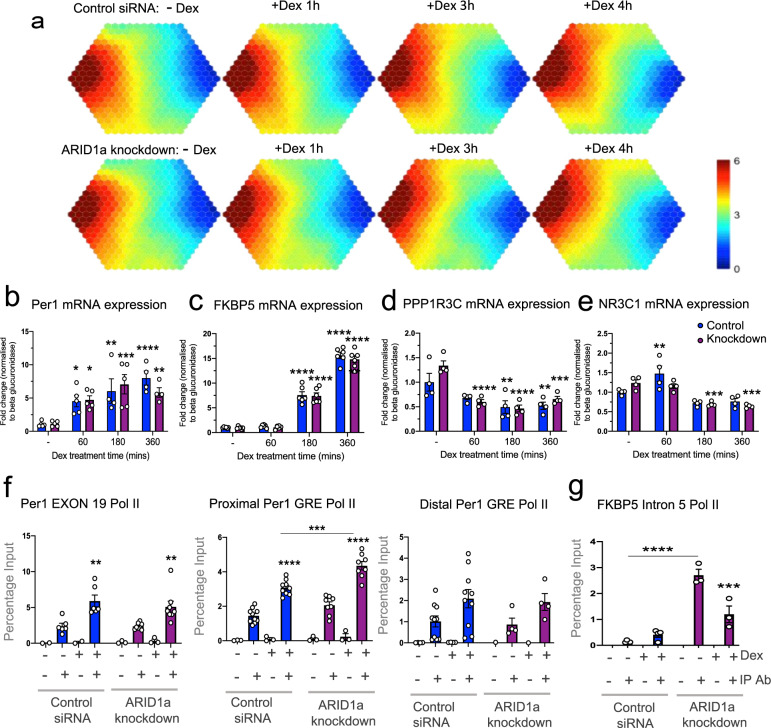
Limited changes in Dex-regulation of target genes with ARID1a knockdown. **a** The supraHex analysis of RNA-Seq data from ARID1a knockdown or control siRNA cells in the absence of Dex (−Dex) and over a Dex treatment timecourse 1, 3, 6 h. In the supra-hexagonal map, genes are hierarchically clustered based on basal expression and the pattern of Dex-regulated change (detailed description in Supplementary Fig. S5). Color key represents mean expression levels (log2 (FPKM data+1) of genes within each hexagon cluster. Control siRNA (‘Control’) and ARID1a knockdown (‘knockdown’) RT-qPCR mRNA validation (with two-way ANOVA results shown) for **b** PER1 (Dex effect *p* < 0.0001; ARID1a knockdown effect *p* = 0.7576; interaction *p* = 0.4572), **c** FKBP5 (Dex effect *p* < 0.0001; ARID1a knockdown effect *p* = 0.7814; interaction *p* = 0.9642), **d** PPP1R3C (Dex effect *p* < 0.0001; ARID1a knockdown effect *p* = 0.1920; interaction *p* = 0.1530), **e** NR3C1 (Dex effect *p* < 0.0001; ARID1a knockdown effect *p* = 0.3279; interaction *p* = 0. 0.0255). RT-qPCR analysis of ChIP assays assessing RNA Polymerase II binding at **f** PER1 Exon 19 (Dex effect *p* < 0.0001; ARID1a knockdown effect *p* = 0.4968; interaction *p* = 0.5281), PER1 proximal GRE (Dex effect *p* < 0.0001; ARID1a knockdown effect *p* < 0.0001; interaction *p* = 0.0551), PER1 distal GRE (Dex effect *p* = 0.0213; ARID1a knockdown effect *p* = 0.7284; interaction *p* = 0.9873) and **g** FKBP5 intron 5 (Dex effect *p* = 0.0057; ARID1a knockdown effect *p* < 0.0001; interaction *p* = 0.0005). Where a significant effect of Dex, siRNA or interaction was found, post-tests were used, and results shown on graphs. For (**b**–**e**) Dunnett’s test results for each Dex treatment timepoint compared to –Dex are shown (*p < 0.05, **p < 0.01, ***p < 0.001, ****p < 0.0001). For (**f**, **g**) results from Bonferroni test results for comparisons between knockdown and control cells, and between −Dex and +Dex are shown on graph (*p < 0.05, **p < 0.01, ***p < 0.001, ****p < 0.0001).

To better understand the extent of loss or functional interference of ARID1a on gene expression, we performed RNA sequencing (RNA-Seq) following a 6 h Dex timecourse in cells with ARID1a knockdown (Supplementary Fig. S1f) or overexpression of the dominant negative ARID1a C-terminal domain (ARID1a-CTD-GFP; Supplementary Fig. S1g) compared to control siRNA (scrambled oligo) or GFP respectively. A bioinformatics hierarchical clustering package [26], supraHex (Fig. 3a and Supplementary Fig. S4a), was used to organize data into groups according to gene expression patterns by training the dataset into clusters based upon basal expression and regulation over time (Supplementary Figs. S5 and S6). This complex analysis clearly demonstrated a striking lack of ARID1a-dependent alterations in the pattern of induced or repressed transcripts. The expression profiles of 936 transcripts (in the ARID1a knockdown experiment) and 353 transcripts (in the ARID1a-CTD-GFP functional interference experiment) were found to be dynamically regulated over time in an ARID1a-independent manner. These included well-characterized and robustly glucocorticoid-inducible or repressible genes (with selected examples shown in Fig. 3b, c; Supplementary Fig. S4b, c, and Fig. 3d e; Supplementary Fig. S4d, e), and many more that were enriched in a diverse range of functional pathways including carbohydrate and fatty acid metabolism, insulin signaling, circadian clock, nuclear receptor transcription pathways, GR regulatory network, developmental biology, inflammatory response, the P53 pathway, DNA repair and cell cycle regulation.

Supporting data from pSer5 RNA Polymerase II ChIP revealed RNA Polymerase II enrichment within candidate glucocorticoid target genes to be largely unaffected by ARID1a knockdown (see example of PER1 exon 19 and distal GRE in Fig. 3f). In fact, in contrast to loss of RNA Polymerase II binding, a significant increase in RNA Polymerase II binding was observed in some cases (see example of PER1 proximal GRE in Fig. 3f). Interestingly, evidence for increased stalling/pausing of pSer5 RNA Polymerase II was found in some target genes after ARID1a knockdown (see example of FKBP5 in Fig. 3g). However, Dex-induced RNA Polymerase II intragenic enrichment was unaffected, consistent with reported findings of increased RNA Polymerase II stalling/pausing without an effect on transcriptional initiation in ovarian clear cell carcinoma cells lacking ARID1a [27].

Importantly, supraHex analysis further revealed a number of transcripts that were differentially expressed in an ARID1a-dependent manner. Basal expression levels of 394 transcripts were significantly altered with ARID1a knockdown, and 174 with ARID1a CTD overexpression. A further 1345 transcripts were differentially regulated over the timecourse when ARID1a was knocked down, while 752 transcripts were differentially regulated over the timecourse when ARID1a-CTD-GFP was overexpressed. Interestingly, many of the ARID1a-dependent differentially expressed transcripts were enriched within a set of related functional pathways and groups, comprising the P53 pathway, DNA repair, and cell cycle regulation (Supplementary Tables S1, S2, and S3 includes detailed information about which genes in these pathways are Dex-regulated in both siRNA control and ARID1a knockdown, in siRNA control only, or in ARID1a knockdown only).

### ARID1a-dependent differential gene expression affects pathways related to P53, DNA damage and repair, and cell cycle

**Fig. 4:**
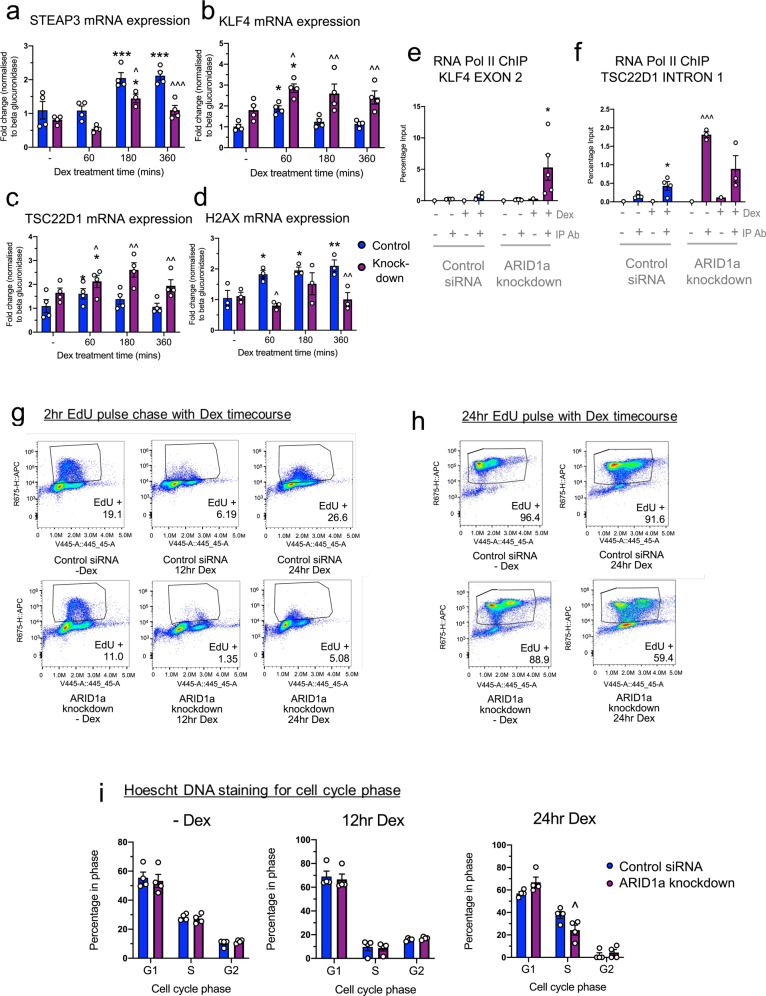
ARID1a knockdown impacts P53-related genes and prolongs Dex-regulated cell cycle G1 arrest. RT-qPCR mRNA validation for **a** STEAP3 (Dex effect *p* < 0.0001; ARID1a knockdown effect *p* < 0.0001; interaction *p* = 0.1145), **b** KLF4 (Dex effect *p* = 0.0055; ARID1a knockdown effect *p* < 0.0001; interaction *p* = 0.6233), **c** TSC22D1 (Dex effect *p* = 0.0458; ARID1a knockdown effect *p* < 0.0001; interaction *p* = 0.4209) and **d** H2AX (Dex effect *p* = 0.0273; ARID1a knockdown effect *p* = 0.0005; interaction *p* = 0.0362). RT-qPCR analysis of ChIP for RNA Polymerase II binding at **e** KLF4 exon 2 (Dex effect *p* = 0.0311; ARID1a knockdown effect *p* = 0.0723; interaction *p* = 0.0627) and **f** TSC22D1 intron 1 (Dex effect *p* = 0.0825; ARID1a knockdown effect *p* < 0.0001; interaction *p* = 0.0054). Where a significant effect of Dex, siRNA or interaction was found, post-tests were used, and results shown on graphs (**a**–**f**). Dunnett’s test results for each Dex treatment timepoint compared to −Dex are shown (*p < 0.05, **p < 0.01, ***p < 0.001). Results from Bonferroni test results for comparisons between knockdown and control cells, and between −Dex and +Dex are shown on graph (^*p* < 0.05, ^^*p* < 0.01, ^^^*p* < 0.001). Flow cytometry analysis **g** shows 2 h of EdU uptake in the absence of Dex (−Dex) and in the final 2 h of a 12 and 24 h Dex timecourse (ie 2 h of EdU uptake between 10–12 and 22–24 h). Flow cytometry analysis **h** shows 24 h of EdU uptake in the presence of Dex (24 h Dex) and in the absence of Dex (−Dex). **i** Hoescht DNA staining in the absence of Dex (−Dex) and in cells harvested at the end of a Dex timecourse (at times 12 and 24 h). (^*p* < 0.05, Two-way ANOVA with Bonferroni test results).

Functional pathway analysis revealed that ARID1a knockdown or functional interference affected both basal expression and Dex-induced regulation of selected genes in the P53 pathway (Fig. 4) while leaving the Dex-induced regulation of several P53 pathway-related genes unaffected (Supplementary Table S1). Notably, Dex-induction was diminished by ARID1a knockdown in several P53 pathway genes, including STEAP3 (Fig. 4a), RGCC, ZNF365, and SESN1 (Supplementary Table S1) which showed Dex-dependent gene induction in control cells but ablated Dex-induction in ARID1a knockdown cells.

Conversely, Dex-induction was increased with ARID1a knockdown in several P53 pathway genes including KLF4 (Fig. 4b), TSC22D1 (Fig. 4c), BTG1, FOS and S100A10 (Supplementary Table S1); KLF4 is a known transcriptional repressor of P53 [28] and TSC22D1 codes for a protein important in protecting P53 from degradation [29]. In support of a direct transcriptional regulation effect on these genes, RNA Polymerase II ChIP analysis of both KLF4 and TSC22D1 showed an increase in Polymerase II binding following ARID1a knockdown (Fig. 4e, f). Dex-dependent gene repression was also affected with a loss of Dex-dependent downregulation in several P53 pathway genes (ATF3, SPHK1, PPP1R15A, RGS16, PDGFA, OSGIN1, and CDKN2AIP) and a gain in Dex-dependent downregulation in others (HSPA4L, SLC7A11, NUPR1, PPM1D, HIST3H2A, CCNG2, and SESN2) (Supplementary Table S1).

Functional pathway analysis also revealed further differential Dex-induction of P53 pathway-related genes with overexpression of the ARID1a-CTD-GFP compared to controls (Supplementary Table S2). These included the P53 effector genes, Jun, BCL2L1, and NDRG1, and P53 pathway-related genes S100A10 and ZFP36L1. Notably, many genes classified as P53-related are also classified with the DNA damage response pathway. Taken together, these changes in functional pathway genes reveal a role for ARID1a in basal and glucocorticoid-mediated regulation of a variety of DNA damage response mechanisms. Interestingly, markers of DNA damage, such as increased expression of several interferons including IFI27, IFI35, IFI6, IFIH1, IFI16, IFI44L, and IFIT5 were further differentially expressed with functional interference of ARID1a, along with the differential basal expression of a group of genes in the PARP family (PARP9, PARP12, and PARP14), which are largely involved in DNA repair [30–32] and cell cycle control [33].

Both GR and P53 have well-characterized roles as facilitators of DNA repair by halting the cell cycle to allow time for the repair machineries to restore genome stability. While many P53-related cell cycle regulators were unaffected by ARID1a knockdown or functional interference, including CDKN1A, GADD45, DDIT4 and SerpinB (full list shown in Supplementary Table S1 and S2), certain other P53-related cell cycle regulators were dysregulated upon either ARID1a knockdown or functional interference. For example, there was an increase in BTG1, a cell cycle regulator that interacts with the P53 pathway [34], and a decrease in Dex-dependent induction of the core histone, H2AX, which is important for P53/P21 mediated cell cycle arrest [35] in knockdown cells (Fig. 4d). Additionally, other histone genes were found to be dysregulated in the absence or functional interference of ARID1a (Supplementary Table S3). Histone gene expression is often restricted to the late G1/early S phase, ensuring their accumulation during DNA replication [36], therefore the expression differences observed may reflect a change in cell cycle progression.

### Prolonged glucocorticoid-dependent G1 arrest in ARID1a knockdown cells

Flow cytometry analysis was used to assess the effect of ARID1a knockdown or functional interference on Dex-induced cell cycle progression and G1 arrest [37–40]. A 2 h EdU treatment, at either 10–12 h or 22–24 h, revealed that the addition of Dex to siRNA control cells promoted cell cycle arrest, observed here between 10 and 12 h of Dex treatment, followed by cell cycle progression observed here between 22 and 24 h (Fig. 4g). When ARID1a was knocked down, Dex was also able to promote G1 arrest, observed between 10 and 12 h of Dex treatment, however, there was less recovery of cell cycle progression observed between 22 and 24 h of Dex treatment. A higher percentage of the ARID1a knockdown cells remained arrested between 22 and 24 h, in comparison to siRNA control cells (Fig. 4g). To additionally compare a full 24 h duration of cellular proliferation in ARID1a knockdown cells versus siRNA control cells, we performed a 24 h EdU incorporation study, either in the absence (−Dex) or presence (24 h) of Dex (Fig. 4h). In the absence of Dex, EdU incorporation was similar in both the ARID1a knockdown and control cells (Fig. 4h). With 24 h Dex treatment, however, EdU uptake was detected in fewer ARID1a knockdown cells than control siRNA cells. Hoechst DNA staining, also included to determine cell cycle phase (Fig. 4i), confirmed that more effective G1 cell cycle arrest occurred during 24 h Dex treatment in ARID1a knockdown cells compared to siRNA control cells.

In stark contrast, overexpression of ARID1a-CTD-GFP had a profound effect on cell cycle progression even in the absence of Dex treatment, indicated by a marked decrease in EdU incorporation in both 2 h EdU pulse and full 24 h EdU incorporation experiments in both the absence and presence of Dex treatment (Supplementary Fig. 7b, c). This suggests that ARID1a-CTD-GFP exerts major effects on the cell cycle independent of GR.

### The DNA damage repair (DDR) pathway is adversely affected by ARID1a knockdown and functional interference

**Fig. 5:**
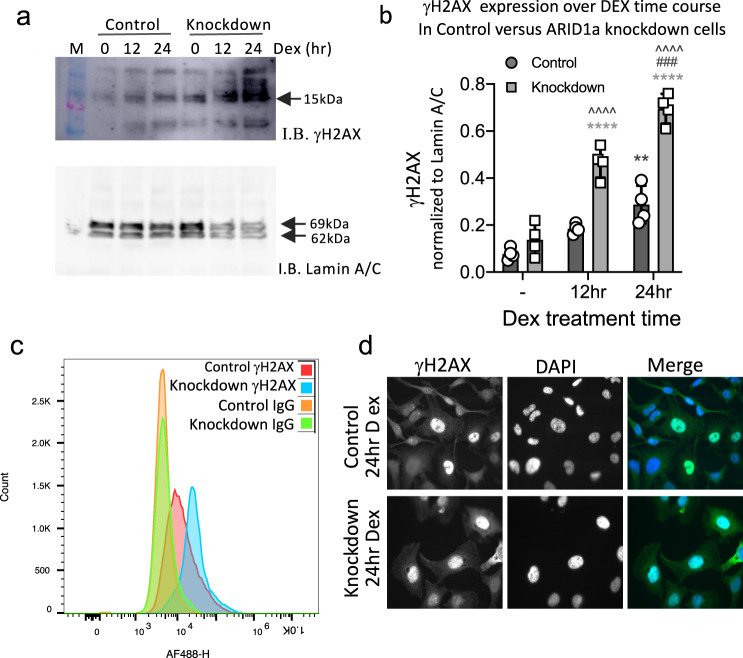
Increased γH2AX during Dex treatment in ARID1a knockdown HeLa cells. **a** γH2AX immunoblot of nuclear extracts prepared from Control siRNA or ARID1a Knockdown HeLa cells revealed increasing levels of γH2AX over the Dex timecourse in both Control and Knockdown HeLa cells. **b** Graph showing Mean ± SEM from densitometry analysis (n4). Two-Way ANOVA reported significant main effects of ARID1a Knockdown (*p* < 0.0001) and Dex treatment (*p* < 0.0001) with a significant interaction (*p* < 0.0001). (*^,#,^^*p* < 0.05, **^,##,^^^*p* < 0.01, ***^,###,^^^^*p* < 0.001, ****^,####,^^^^^*p* < 0.0001) are indicated on graph: *Significant difference relative to time 0; ^#^Significant difference between 12 h and 24 h Dex treatment; ^Significant difference between ARID1a Knockdown and Control at matched times. **c** Flow cytometry analysis of 24 h Dex treated cells stained with rabbit anti-γH2AX, secondary anti-rabbit AF488. Isotype matched rabbit IgG, secondary anti-rabbit AF488 indicates background fluorescence. **d** Immunofluorescent images of 24 h Dex treated Control siRNA and ARID1a Knockdown Hela cells stained with rabbit anti-γH2AX, secondary anti-rabbit AF488.

In order to assess whether DNA damage spontaneously accumulated over time during the Dex treatment timecourse, we assessed levels of phospho-Serine139 Histone H2AX (γH2AX; a biomarker for DNA damage [41, 42]) in the absence of Dex (−Dex), and at 12 and 24 h of Dex treatment. These experiments were performed in the absence of genotoxic agents or physical DNA damage induction protocols such as ultraviolet or ionizing radiation. Western blot analysis revealed that γH2AX levels increased over the Dex timecourse in both ARID1a knockdown and siRNA control cells (Fig. 5a, b). However, γH2AX levels were significantly increased in the ARID1a knockdown cells, most markedly at the 24 h Dex treatment timepoint. Flow cytometry analysis of cells stained with the anti-γH2AX and Alexa-fluor 488 secondary antibody also showed significantly greater γH2AX accumulation at 24 h of Dex treatment in ARID1a siRNA knockdown compared to siRNA controls (Fig. 5c). Immunofluorescence studies qualitatively confirmed heterogeneous γH2AX+ expression in the cells, with more γH2AX+ cells in the ARID1a knockdown population compared to siRNA controls, shown here at the 24 h Dex treatment timepoint (Fig. 5d).

In contrast, cells transfected with ARID1a-CTD-GFP exhibited the highest levels of γH2AX (assessed by Flow cytometry analysis of cells immuno-stained with anti-γH2AX and Alexa-fluor 647 secondary antibody) in the absence of Dex, when compared to matched GFP+ control cells (Supplementary Fig. S7a). Significantly higher levels of γH2AX were also detected in the ARID1a-CTD-GFP expressing cells at 12 and 24 h Dex treatment, when compared to matched GFP+ control cells. Taken together, this indicated that ARID1a-CTD-GFP had significant effects on the accumulation of spontaneously arising DNA damage, independent of glucocorticoids. Interestingly, the EdU incorporation analysis revealed that ARID1a-CTD-GFP overexpression significantly reduced cellular proliferation in the absence of Dex, as well as during Dex treatment (Supplementary Fig. S7b). Taken together, these data strongly suggest that Dex treatment of the ARID1a-CTD-GFP cells was able to reduce accumulation of spontaneously arising DNA damage, potentially mediated via other DNA repair proteins, such as PARP9, PARP12, and PARP14, which were found to be upregulated in the ARID1a-CTD-GFP cells (Supplementary Fig. S8).

### ARID1a is required for GR interactions with cell cycle regulators and DNA repair proteins

**Fig. 6:**
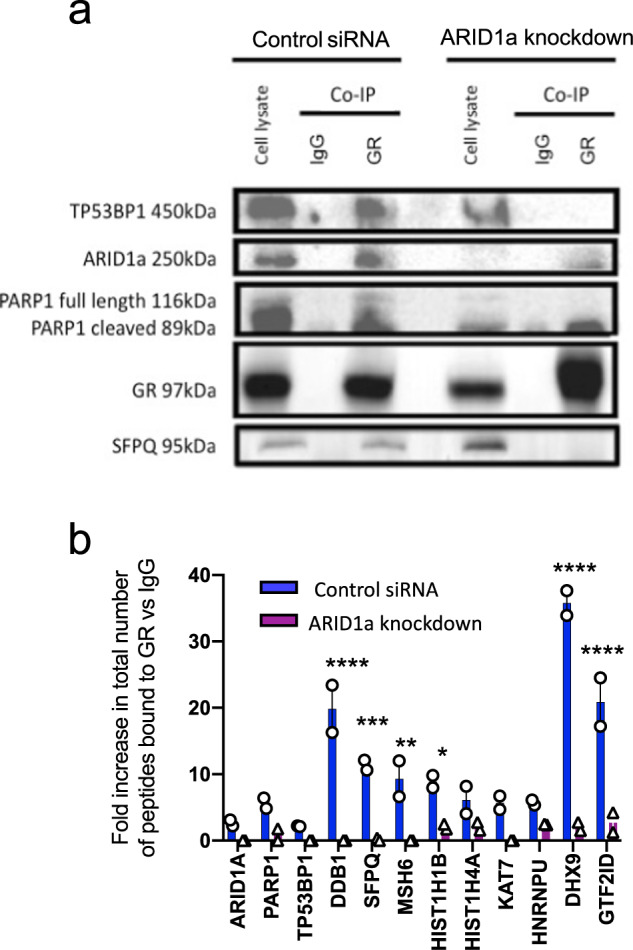
Chromatin-associated GR co-immunoprecipitation data using liquid chromatography–mass spectrometry (LC–MS). **a** Western blot showing GR co-immunoprecipitated proteins from ARID1a knockdown and control siRNA HeLa cells, both treated with 100 nM Dex for 30 min. **b** LC–MS results show loss of several proteins (>80 kDa) bound to GR (FDR < 0.01) resulting from ARID1a knockdown, including PARP1, P53BP1, DDB1, SFPQ, MSH6, Histone Cluster 1 H1 Family Member B (HIST1H1B), Histone Cluster 1 H4 Family Member A (HIST1H4A), KAT7, Heterogeneous Nuclear Ribonucleoprotein U (HNRNPU), ATP-Dependent RNA Helicase A (DHX9) and General transcription factor II isoform D (GTF2ID). Two-way ANOVA found a significant effect of antibody relative to IgG (*p* < 0.0001), siRNA (*p* < 0.0001), and interaction (*p* < 0.0001) Bonferroni test results for significant difference in GR-bound peptides in control versus ARID1a knockdown are shown on graph (*p < 0.05, **p < 0.01, ***p < 0.001, ****p < 0.0001).

Liquid chromatography–mass spectrometry (LC–MS) was used to identify GR-interacting proteins in control cells compared to when ARID1a was knocked down, in order to identify an ARID1a-dependent GR protein complex at the DNA template. These data confirmed an interaction between GR and ARID1a in control cells and further identified ARID1a-dependent GR interactions with key P53-related and DNA repair proteins (Fig. 6a, b, Supplementary Table S4) including P53 binding protein 1 (P53BP1), a protein important for DNA damage repair [43] as well as P53 mediated transcriptional activation [44].

Other ARID1a-dependent GR interactions included PARP1, a protein required for DNA repair and linked to regulation of P53 mediated cell cycle arrest [45], and DNA damage-binding protein 1 (DDB1), a protein important for recognizing and initiating repair of DNA lesions [46]. DDB1 has also been linked to the P53 pathway, with its loss linked to P53 pathway activation [47–49]. ARID1a knockdown also resulted in loss of GR interaction with the histone acetyltransferase, lysine acetyltransferase 7 (KAT7), and the splicing factor proline and glutamine-rich (SFPQ) protein which is known to be required for RNA processing and transport [50] and more recently found in a complex involved in DNA repair [51, 52].

In the functional interference study, ARID1a-CTD-GFP was found to interact with GR resulting in the loss of endogenous ARID1a binding (Supplementary Fig. S9). Here we also report a loss of GR interaction with P53BP1, PARP1, DDB1, and KAT7, indicating that overexpression of ARID1a-CTD alone interferes with the interaction between GR and numerous DNA repair proteins.

## Discussion

Despite previous reports that ARID1a (BAF250) is linked to glucocorticoid resistance [4] and is widely mutated across a multitude of human carcinomas [5–9], its precise role in GR signaling has not been well characterized. As ARID1a interacts with GR [12, 13] and is a component of the hSWI/SNF (BAF) complex, we and others have previously speculated that ARID1a deletion or mutation may cause large-scale dysregulation of glucocorticoid-target genes [4, 12, 13] potentially by impairing GR interactions with the BAF complex [21] and reducing chromatin accessibility for the transcriptional machinery and RNA Polymerase II [17]. To the contrary, we found that ARID1a appeared to play a greater role in pre-setting the chromatin architecture, potentially at BRG1-dependent sites [53], than in mediating glucocorticoid-dependent transcriptional regulation. There was a striking lack of global change in RNA expression for the vast majority of glucocorticoid-target genes when ARID1a was knocked down or subjected to functional interference. Consistent with this finding, regulatory elements for inducible and repressible glucocorticoid-target genes still possessed significant Dex-induced GR binding despite ARID1a knockdown.

ATAC-Seq data revealed that chromatin pre-accessibility was significantly affected by ARID1a knockdown, with a significant decrease in pre-accessibility at a large number of sites, and a significant increase at a smaller number of sites. Interestingly, a small but notable subset of sites were Dex-inducible in both ARID1a knockdown and control cells, and many of these were within loci containing genes found to be similarly Dex-regulated in control siRNA and ARID1a knockdown cells in the RNA-Seq analysis. Examples of genes that were in loci found to undergo Dex-inducible chromatin remodeling and Dex-dependent mRNA induction, in both ARID1a knockdown and control cells, included KLF9 [54, 55], DDIT4 [56], GADD45a [57], and SERPINB [58, 59], which are all important for cell proliferation and/or cell cycle regulation and have therefore also been grouped together with P53-regulated pathways, again supporting our conclusions of a selective effect of ARID1a knockdown.

STEAP3 is one of the selectively affected genes identified in our analyses. The lack of chromatin pre-accessibility at sites in this gene may explain the subsequent impairment in its Dex-induced transcriptional regulation in the ARID1a knockdown cells. As an ARID1a-dependent glucocorticoid target, impaired Dex-dependent transcriptional regulation of STEAP3 has considerable functional significance for loss or mutation of ARID1a, as it has been speculated to play a role downstream of P53 to control cell cycle progression. Interestingly, in addition to STEAP3, only a small number of other genes were identified by RNA-Seq analysis as dysregulated by ARID1a knockdown or functional interference, yet according to functional pathway analysis, these were also enriched in the P53, DNA repair, and cell cycle regulatory pathways. Despite the large numbers of ‘so-called’ P53-related genes that were found to be differentially regulated in our study, HeLa cells lack a stable P53 protein [60]. Therefore, our results now demonstrate that many of the P53-associated cell cycle and DNA repair pathways are regulated in the absence of P53. Furthermore, we have identified a protein complex comprising GR, ARID1a and P53BP1. P53BP1 is a well-characterized P53 cofactor, and the contribution of P53BP1 to transcriptional regulation of the DNA damage checkpoint has been reported to be P53-dependent yet its direct role in DNA damage repair is P53-independent [61, 62]. Consistent with the latter, here we report that the GR-ARID1a-P53BP1 complex plays an important functional role in DNA damage repair in the absence of P53. Our findings are consequently particularly relevant to P53 null cancers, which are prevalent due to P53 being the most commonly altered gene in human carcinomas [10, 11].

### Cell cycle regulation and DNA damage repair

Glucocorticoids are known to mediate cell cycle arrest [38–40, 63, 64], but it is important to note that ARID1a is well documented as an essential regulator of cell cycle arrest in its own right [63, 65] independent of Dex treatment. Here we show that ARID1a is not required for Dex-induced cell cycle arrest. Both ARID1a knockdown and siRNA control cell populations were similarly asynchronously progressing through the cell cycle in the absence of Dex, then were similarly G1 arrested by Dex at 12 h treatment time. Interestingly, at 24 h of Dex treatment, a greater proportion of ARID1a knockdown cells remained in G1 phase, with significantly fewer cells progressing to S phase compared to siRNA control cells. Similarly, Prekovic et al. found reduced cellular proliferation due to impaired cell cycle progression in ARID1a mutated non-lymphoid cancers treated with glucocorticoids [66].

As DNA damage repair (DDR) and cell cycle regulation are intrinsically linked, the prolonged cell cycle arrest observed in ARID1a knockdown cells may be related to the increased DNA damage we have characterized in the ARID1a knockdown cells. ARID1a has previously been shown to be important for DNA damage repair and checkpoint regulation, with ARID1a interacting with AT Rich sequences in DNA and being recruited to DNA strand breaks [14]. No genotoxic agent or physical damage was used in our experiments, so the observed accumulation of DNA damage likely represents spontaneously arising DNA breaks, which are known to occur during the transcriptional response to steroid hormones such as Dex [67, 68], as well as during DNA replication during cell division [69]. Consistent with this, we detected a significant increase in DNA damage in ARID1a knockdown cells by assessing the levels of γH2AX, a biomarker of genomic instability, and indicative of DNA damage [41, 42]. γH2AX also plays a critical structural role in the repair process, specifically contributing to the efficient recruitment and retention of remodeling factors at the DDR site, notably including P53BP1 [70]. Once DDR has efficiently proceeded, levels of γH2AX reduce again, in a mechanism previously demonstrated to involve H2AX dephosphorylation by protein phosphatase 2A [71, 72]. Thus, γH2AX has proved to be a sensitive method of monitoring the progression of DNA damage and repair. The increased γH2AX levels over the Dex timecourse is therefore consistent with impaired GR-ARID1a mediated DDR in ARID1a knockdown cells compared to control siRNA cells.

Increased DNA damage was also observed in ARID1a-CTD-GFP cells compared to GFP control cells. However, in contrast to the ARID1a knockdown cells, the highest levels of γH2AX in the ARID1a-CTD-GFP overexpressing cells were detected in the absence of Dex. Consistent with this finding, there was also an effect of ARID1a-CTD-GFP overexpression on cell cycle that was most pronounced in the absence of Dex. Levels of γH2AX then reduced significantly with Dex treatment, indicating that other DNA repair proteins may play a role. Accordingly, the DNA repair proteins PARP9, PARP12 and PARP14 had increased basal expression in the ARID1a-CTD-GFP cells but not in the ARID1a knockdown cells, representing a major point of difference between the two experimental models.

Our RNA-Seq data found some striking similarities between the effects of ARID1a knockdown and functional interference experiments, with significantly decreased expression in several DNA repair genes in response to Dex when ARID1a is knocked down or functionally interfered with. Additionally, the data revealed further indications of DNA damage with dysregulation of several interferon genes. Our proteomics data provided new insights into GR’s role in this mechanism, with the identification of a macromolecular complex comprising GR, ARID1a and several proteins involved in DNA repair. The P53 binding protein, P53BP1, detected in the ARID1a-dependent complex with GR, is well known for its multiple roles in the DNA damage response, including promoting checkpoint signaling and acting as a scaffold for recruitment of DNA damage response proteins to damaged chromatin [73–78]. Also identified in the ARID1a-dependent GR complex, MSH6 is part of the MutS alpha complex that binds to DNA mismatches to initiate DNA repair, and DDB1 is involved in both DNA repair and protein ubiquitination.

Furthermore, identification of PARP1, which is important for DNA repair, as well as the regulation of chromatin structure and specific gene expression [79, 80], cell death, and mitosis [81] provided strong evidence that our identified protein complex may act to integrate multiple functions of transcriptional control, DNA damage recognition and repair, and cell cycle checkpoint controls, all at the chromatin template. Consistent with this, SFPQ was also detected in the ARID1a-dependent protein complex with GR. SFPQ plays a role in almost every step of gene regulation, from DNA repair to transcriptional regulation, RNA processing, and transport, as well as being part of a larger family described as acting as a multipurpose molecular scaffold [50].

The presence of the histone acetyltransferase KAT7/Myst2/HBO1 in the ARID1a-dependent GR protein complex revealed further mechanistic insight. In addition to KAT7’s established role in steroid-dependent transcription, demonstrated for the androgen receptor (AR) [82], the progesterone receptor (PR) [83] and now GR, it is known to play a crucial role in nucleotide excision repair, acetylating histones at sites of DNA damage to facilitate recruitment of the DNA damage recognition and repair factor XPC [84]. In addition, KAT7 interacts with various candidate tumor suppressors [85] although the KAT7-ARID1a interaction has not been reported until now.

Taken together, the GR-ARID1a complex we have identified is well placed and adequately prepared to act as a sensor of cellular stress and/or damage to maintain the balance between genome integrity and cell cycle progression. The components of the complex are also druggable targets; PARP inhibitors are already being used as a treatment for women with ovarian or fallopian tube cancers [14, 51, 86] and may have a benefit in P53 null and/or ARID1a null cancers which are glucocorticoid resistant. Similarly, many of the other components identified in the GR-ARID1a macromolecular complex may prove to be useful cancer therapy targets. Particular interest is being shown in the development of new anti-cancer therapeutics which target KAT7/HBO1 [87] and the closely related KAT6A/B by inhibiting their HAT enzymatic activity [88] while SFPQ is speculated to be a particularly useful cancer therapy target for inactivating multiple pathways in cancer cells resistant to other chemotherapy options [89, 90].

Finally, in light of our findings, the importance of assessing DNA damage status and cell cycle arrest when deciding on treatment of different cancers becomes more pertinent. For example, in the case of the ARID1a knockdown cells, increased DNA damage was sufficient to initiate and prolong G1 arrest. However, many cell types including the HeLa cells used in our study, and the non-small cell lung cancers used in the study by Prekovic et al. [66], do not undergo apoptosis but instead become ‘dormant’ as a consequence of glucocorticoid treatment, and this is often reversible. In cases when the cancer cells do not undergo programmed cell death, it may be beneficial to use a chemical manipulation of the mechanisms already in place, in order to directly promote cell death. New therapeutic strategies and tools, for example, drugs that force cells with DNA damage to bypass cell cycle checkpoints through S and G2/M arrest and enter mitosis, leading to cell death by mitotic catastrophe [91], look promising as a substantial improvement to cancer therapy.

## Methods

### siRNA and plasmids

Four siRNAs targeting ARID1a and a non-targeting scrambled oligo control were obtained from Dharmacon (GE healthcare). ARID1a-CTD was constructed from 2052 bp of the 3′ portion (4899–6855 bp) from pcDNA6-ARID1a (Addgene; 39311).

### Cell line

HeLa cells obtained from the European Collection of Cell Cultures (ECACC; Sigma-Aldrich) were maintained in Dulbecco’s Modified Eagle Medium (DMEM) with L-glutamine and glucose, 10% fetal calf serum, in a humidified incubator (LEEC Ltd, UK) at 37 °C and 5% CO_2_.

### Transfections

For ARID1a knockdown, cells were transfected with ARID1a siRNA. The control comparison cells (siRNA control) were transfected with non-targeting oligonucleotides (Supplementary Fig. S1 and Table S5) using Lipofectamine siRNA/imax (Invitrogen, UK). For ARID1a functional interference studies (Supplementary Fig. S1d), cells were transfected with 500 ng/μl of the ARID1a-CTD-GFP construct (Supplementary Fig. S1d) using Lipofectamine 3000 (Invitrogen, UK).

### Chromatin Immunoprecipitation

Chromatin immunoprecipitation (ChIP) assays were performed as previously described [24]. Soluble chromatin was prepared using MNase digestion [50 mM Tris-HCl, pH 7.5, 4 mM MgCl_2_, 1 mM CaCl_2_, 0.32 mM sucrose, 2 mM NaF, 0.2 mM NaVan] with 2 units of MNase (Sigma, UK)/180 μg chromatin, then immunoprecipitated with H300 GR antibody (Santa Cruz; sc-8992), RNA Polymerase II (phosphoserine-5) antibody (ACTIVE MOTIF; 39233) or rabbit non-immune serum (Santa Cruz; sc-2027). For sequencing, libraries were prepared from two replicates/condition. RT-qPCR was performed using SYBR reagents (Applied Biosystems) and primers described in [24] (Supplementary Table S6).

### ATAC-Seq

HeLa cells were detached from flasks using Accutase, then resuspended in PBS at 6 million cells/ml. Nuclei were isolated and ATAC was performed following methods previously described [92].

### RNA isolation

Cells were collected in TRIzol (Ambion, Life Technologies, UK), and total RNA was purified using membrane columns (RNAeasy minikit, Qiagen, UK). An on-column DNase step (Qiagen, UK) was used to ensure the removal of genomic DNA. RNA was eluted in nuclease-free water, and stored at −80 °C.

### Quantitative real-time PCR

RT-qPCR was carried out on reverse complementary DNAs to determine the approximate levels of mRNA transcripts in RNA samples. Experiments were performed several times with at least an *n* of 3 for each sample. PCR primer sequences used are listed in Supplementary Table S7 (mRNA primers).

### RNA-Seq

Raw data from high-throughput RNA sequencing was uploaded to Galaxy bioinformatics (www.galaxyproject.org). Three lanes were uploaded for each RNA sample. For each condition an n of at least 3 were assessed. A workflow was run for each lane, forward (dataset 1) and reverse strand (dataset 2). The Cuff Diff parameters included were geometric library organization, pooled dispersion estimation, 0.05 false discovery rate, Min alignment count 10, multi-read correct, bias correction, and cuff-links effective length correction.

### Nuclear extracts and Western blotting

Cells were collected, and nuclear extracts prepared as previously described [24, 93, 94]. Both nuclear and cytoplasmic samples were separated on a 5–7.5% SDS polyacrylamide gel (SDS-PAGE) (then transferred to a Polyvinylidene fluoride membrane (PVDF) membrane (GE Healthcare, UK).

### Fluorescence-activated cell sorting

#### Immunostaining for phospho-Ser139 Histone H2A.X (γH2AX)

Cells were detached from plates in Accutase. 1 × 10^6^ cells were fixed in 4% formaldehyde then permeabilized with 90% methanol. Following washes in PBS then incubated with rabbit anti-γH2AX (2577; Cell Signaling Technology) or concentration-matched Rabbit (DA1E) mAb IgG XP^®^ Isotype Control (3900; Cell Signaling Technology) for 1 h, followed by incubation with either Goat anti-Rabbit IgG Secondary Antibody, Alexa Fluor™ 488 (A11008; Invitrogen) or Goat anti-Rabbit IgG Secondary Antibody, Alexa Fluor™ Plus 647 (A32733; Invitrogen). Cells were fixed in 1% formaldehyde and resuspended in 1 x PBS to be analyzed on a novocyte flow cytometer. Cells were analyzed using FlowJo software (TreeStar Software, USA).

### Click-iT™ Plus EdU flow cytometry assay

EdU reagent (from kit C10634; Invitrogen) was added for the final 2 h of the Dex timecourse (10–12 h and 22–24 h, in the case of the 12 and 24 h Dex treatments respectively) and for 2 h in the absence of Dex (in the case of –Dex treatment) according to the manufacturer’s recommended instructions. Cells were detached using Accutase (A1110501, Gibco™) prior to processing with Click-iT™ Plus EdU Flow Cytometry Assay Kit (C10634; Invitrogen). Cells were fixed in 1% formaldehyde and resuspended in 1× PBS before being analyzed on a Novocyte flow cytometer, using FlowJo software (TreeStar Software, USA). Data shown is from one typical representative experiment analyzing 3000 cells. All Click-it Plus experiments were repeated a minimum of three times, for three biological repeats.

### Co-immunoprecipitation and liquid chromatography–mass spectrometry (LC–MS)

Cells were washed and collected in ice-cold PBS with phosphatase inhibitors. Cell suspension was centrifuged for 5 min at 500 × *g* at 4 °C. The cell pellet was resuspended in hypotonic S1 buffer and incubated on ice for 15 min to isolate the nuclei. The nuclear fraction was digested using an Enzymatic shearing cocktail (ActiveMotif, UK) on ice for 90 min. EDTA was added to stop the reaction. The nuclear fraction was collected and added to a buffer [150 mM NaCl, 50 mM Hepes, 5 mM MgCl_2_, 0.1 mM EDTA, 1% Igepal] supplemented with DTT, protease, and phosphatase inhibitors, for co-immunoprecipitation with a GR antibody (H300, Santa-Cruz) or rabbit IgG control (2729, Cell Signaling Technology, UK). Nuclear fractions were incubated with antibody overnight at 4 °C. Protein A magnetic beads were added for 3 h and then the beads were washed, and complexes eluted in 1× Laemmli buffer supplemented with 100 mM DTT. For LC–MS, size selection was performed so that only proteins of 80 kDa or larger were detected using LC–MS. Replicate co-immunoprecipitation experiments were performed for validation by western blot.

### Antibodies

Antibodies used for western blotting were: ARID1a/BAF250 A301-040A (Bethyl labs, 1:1000), ARID1a/BAF250 A301-041A (Bethyl labs, 1:5000), GR E20 (Santa Cruz, sc-1003, 1:500), GR D6H2L (Cell Signaling Technology, 1:1000), γH2AX (#2577; Cell Signaling Technology), PARP1 46D11 (Cell Signaling Technology), 53BP1 4937 (Cell Signaling Technology) and SFPQ ab38148 (Abcam), Anti Beta Tubulin T4026 (Sigma) or Anti Lamin A/C 2032 (Cell Signaling Technology, 1:1000). Secondary antibodies used were, ECL anti-rabbit, NA934V (GE Healthcare, UK, 1:10,000), or ECL anti-mouse, NA931V (GE Healthcare, UK, 1:10,000).

### Statistical analysis - ChIP-Seq analysis

Quality of sequencing was assessed using FastQC (usegalaxy.org) and sequences were trimmed to 36 bases. Single end reads were uniquely aligned to the human genome assembly (hg19) using Bowtie2 and PCR duplicates removed (SAMtools v1.3.1). Counts were normalized to 10 million tags to allow for cross-sample comparisons and visualized using the UCSC genome browser (https://genome.ucsc.edu/). Enrichment of GR ChIP-Seq to 1% input control, were identified using findPeaks (HOMER v4.9.1) at relaxed settings (-F1 -L1 -P.1 -LP.01 -poisson .1 -style factor). Of these, only concordant replicate enrichments that passed an estimated irreproducible discovery rate threshold were kept for downstream analysis (IDR v2.0.3 – set to 0.01). Any concordant enrichments that overlapped were merged into a single region using mergePeaks (HOMER v4.9.1) and raw input ChIP-Seq tag counts were subtracted from corresponding GR tag counts using annotatePeaks.pl. Differential fold change between all conditions was assessed using getDiffExpression.pl (HOMER v4.9.1) and DESeq2 [95]. For DESeq2 analysis, tags were normalized to total tags within enriched regions and considered significantly changed to no Dex control (as well as between treatments) if FDR < 0.05, fold change > 0.585 (Log2) and *p* value <0.05 adjusted for multiple comparisons. All other comparisons were given the value 0. All enrichment regions were annotated with annotatePeaks.pl (HOMER v4.9.1) to closest transcriptional start site (TSS) (UCSC RefSeq GCD_000001405.25_GCCh37.p13 (2017-04-19)). Fold change (Log2) between treatments was visualized using heatmap.2 (RStudio 1.0.153, RStudio Team (2016). RStudio: Integrated Development for R. RStudio, Inc., Boston, MA URL http://www.rstudio.com/), GraphPad Prism v6.07 for Windows (La Jolla, CA, USA, www.graphpad.com) and Venny v2.1 (http://bioinfogp.cnb.csic.es/tools/venny/index.html).

### ATAC-Seq analysis

ATAC-Seq peaks were called on each sample separately using the MACS algorithm [96] and filtered by excluding those with tag density less than the median tag density in each sample. These remaining peaks were merged and combined to generate a list of non-overlapping peaks. ATAC-Seq tag densities corresponding to the peaks were quantified by running the annotatePeaks.pl program in the HOMER suite [97]. Finally, we obtained a list of 101,807 peaks encompassing 8 ATAC-Seq samples. DESeq [98] was used to identify differentially open peaks on 6 possible pairs of comparisons. ATAC-Seq peaks with FDR-adjusted *p* value < 0.05 and log2 |Fold Change| > 1 in at least one comparison were considered DORs between samples, and displayed in the z-score heatmap (in Fig. 2a). Negative binomial distribution was fitted to evaluate the significance of ±Dex and ARID1a knockdown effects and calculate *p* values. 4567 DORs were identified in total. K-means algorithm was used to reveal the clusters within the DOR with z-score.

The tag density heatmap (Supplementary Fig. S3) was generated over ±1 kb base pairs region around the center of the ATAC-Seq peak using an in-house R package from the tag density profiles generated by the Homer suite. Tag density signals were transformed in a log2 scale.

### RNA-Seq analysis

For RNA-seq data differential gene expression was calculated as fragments per kilobase per million mapped reads (FPKM) values; summed fragments of each transcript with same gene ID. After Benjamini–Hochberg false discovery correction, genes with adjusted p values less than 0.05 were considered as differentially expressed genes [99]. The supraHex analysis [26] was used to aid visualization of regulation patterns in the data.

Graphpad Prism Version 6 (Graphpad software inc., USA) was used to analyze RT-qPCR data by Two-way ANOVAs with a Dunnett’s or Bonferroni post hoc test. For each result, the statistical test used is stated. The mean and standard error of the mean (SEM) values were always calculated.

## Supplementary information

Supplementary figures

## Data Availability

All raw and processed sequencing data generated in this study have been submitted to the NCBI Gene Expression Omnibus (GEO; https://www.ncbi.nlm.nih.gov/geo/) under accession number GSE207411.

## References

[1] Herr I, Pfitzenmaier J (2006). Glucocorticoid use in prostate cancer and other solid tumours: implications for effectiveness of cytotoxic treatment and metastases. Lancet Oncol.

[2] Planey SL, Litwack G (2000). Glucocorticoid-induced apoptosis in lymphocytes. Biochem Biophys Res Commun.

[3] Rutz HP (2002). Effects of corticosteroid use on treatment of solid tumours. Lancet.

[4] Pottier N, Yang W, Assem M, Panetta JC, Pei D, Paugh SW (2008). The SWI/SNF chromatin-remodeling complex and glucocorticoid resistance in acute lymphoblastic leukemia. J Natl Cancer Inst.

[5] Guan B, Wang TL, Shih Ie M (2011). ARID1A, a factor that promotes formation of SWI/SNF-mediated chromatin remodeling, is a tumor suppressor in gynecologic cancers. Cancer Res.

[6] Mamo A, Cavallone L, Tuzmen S, Chabot C, Ferrario C, Hassan S (2012). An integrated genomic approach identifies ARID1A as a candidate tumor-suppressor gene in breast cancer. Oncogene.

[7] Zang ZJ, Cutcutache I, Poon SL, Zhang SL, McPherson JR, Tao J (2012). Exome sequencing of gastric adenocarcinoma identifies recurrent somatic mutations in cell adhesion and chromatin remodeling genes. Nat Genet.

[8] Helming KC, Wang X, Wilson BG, Vazquez F, Haswell JR, Manchester HE (2014). ARID1B is a specific vulnerability in ARID1A-mutant cancers. Nat Med.

[9] Wiegand KC, Shah SP, Al-Agha OM, Zhao Y, Tse K, Zeng T (2010). ARID1A mutations in endometriosis-associated ovarian carcinomas. N Engl J Med.

[10] Shain AH, Pollack JR (2013). The spectrum of SWI/SNF mutations, ubiquitous in human cancers. PLoS One.

[11] Muller PA, Vousden KH (2013). p53 mutations in cancer. Nat Cell Biol.

[12] Inoue H, Furukawa T, Giannakopoulos S, Zhou S, King DS, Tanese N (2002). Largest subunits of the human SWI/SNF chromatin-remodeling complex promote transcriptional activation by steroid hormone receptors. J Biol Chem.

[13] Nie Z, Xue Y, Yang D, Zhou S, Deroo BJ, Archer TK (2000). A specificity and targeting subunit of a human SWI/SNF family-related chromatin-remodeling complex. Mol Cell Biol.

[14] Shen J, Peng Y, Wei L, Zhang W, Yang L, Lan L (2015). ARID1A Deficiency Impairs the DNA Damage Checkpoint and Sensitizes Cells to PARP Inhibitors. Cancer Discov.

[15] John S, Sabo PJ, Thurman RE, Sung MH, Biddie SC, Johnson TA (2011). Chromatin accessibility pre-determines glucocorticoid receptor binding patterns. Nat Genet.

[16] Cordingley MG, Riegel AT, Hager GL (1987). Steroid-dependent interaction of transcription factors with the inducible promoter of mouse mammary tumor virus in vivo. Cell.

[17] Fryer CJ, Archer TK (1998). Chromatin remodelling by the glucocorticoid receptor requires the BRG1 complex. Nature.

[18] Archer TK, Zaniewski E, Moyer ML, Nordeen SK (1994). The differential capacity of glucocorticoids and progestins to alter chromatin structure and induce gene expression in human breast cancer cells. Mol Endocrinol.

[19] Biddie SC, John S, Sabo PJ, Thurman RE, Johnson TA, Schiltz RL (2011). Transcription factor AP1 potentiates chromatin accessibility and glucocorticoid receptor binding. Mol Cell.

[20] Grontved L, John S, Baek S, Liu Y, Buckley JR, Vinson C (2013). C/EBP maintains chromatin accessibility in liver and facilitates glucocorticoid receptor recruitment to steroid response elements. EMBO J.

[21] Hoffman JA, Trotter KW, Ward JM, Archer TK (2018). BRG1 governs glucocorticoid receptor interactions with chromatin and pioneer factors across the genome. Elife.

[22] Swinstead EE, Miranda TB, Paakinaho V, Baek S, Goldstein I, Hawkins M (2016). Steroid receptors reprogram FoxA1 occupancy through dynamic chromatin transitions. Cell.

[23] Trotter KW, Fan HY, Ivey ML, Kingston RE, Archer TK (2008). The HSA domain of BRG1 mediates critical interactions required for glucocorticoid receptor-dependent transcriptional activation in vivo. Mol Cell Biol.

[24] Stubbs FE, Birnie MT, Biddie SC, Lightman SL, Conway-Campbell BL (2018). SKOV3 cells containing a truncated ARID1a protein have a restricted genome-wide response to glucocorticoids. Mol Cell Endocrinol.

[25] Pooley JR, Flynn BP, Grontved L, Baek S, Guertin MJ, Kershaw YM (2017). Genome-wide identification of basic helix-loop-helix and NF-1 MOtifs Underlying GR binding sites in male rat hippocampus. Endocrinology.

[26] Fang H, Gough J (2014). supraHex: an R/Bioconductor package for tabular omics data analysis using a supra-hexagonal map. Biochem Biophys Res Commun.

[27] Trizzino M, Barbieri E, Petracovici A, Wu S, Welsh SA, Owens TA (2018). The tumor suppressor ARID1A controls global transcription via pausing of RNA polymerase II. Cell Rep.

[28] Rowland BD, Bernards R, Peeper DS (2005). The KLF4 tumour suppressor is a transcriptional repressor of p53 that acts as a context-dependent oncogene. Nat Cell Biol.

[29] Yoon CH, Rho SB, Kim ST, Kho S, Park J, Jang IS (2012). Crucial role of TSC-22 in preventing the proteasomal degradation of p53 in cervical cancer. PLoS ONE.

[30] Ame JC, Rolli V, Schreiber V, Niedergang C, Apiou F, Decker P (1999). PARP-2, A novel mammalian DNA damage-dependent poly(ADP-ribose) polymerase. J Biol Chem.

[31] de Murcia G, Menissier de Murcia J (1994). Poly(ADP-ribose) polymerase: a molecular nick-sensor. Trends Biochem Sci.

[32] Petrucco S (2003). Sensing DNA damage by PARP-like fingers. Nucleic Acids Res.

[33] Vyas S, Chesarone-Cataldo M, Todorova T, Huang YH, Chang P (2013). A systematic analysis of the PARP protein family identifies new functions critical for cell physiology. Nat Commun.

[34] Cortes U, Moyret-Lalle C, Falette N, Duriez C, Ghissassi FE, Barnas C (2000). BTG gene expression in the p53-dependent and -independent cellular response to DNA damage. Mol Carcinog.

[35] Fragkos M, Jurvansuu J, Beard P (2009). H2AX is required for cell cycle arrest via the p53/p21 pathway. Mol Cell Biol.

[36] Osley MA (1991). The regulation of histone synthesis in the cell cycle. Annu Rev Biochem.

[37] Goya L, Maiyar AC, Ge Y, Firestone GL (1993). Glucocorticoids induce a G1/G0 cell cycle arrest of Con8 rat mammary tumor cells that is synchronously reversed by steroid withdrawal or addition of transforming growth factor-alpha. Mol Endocrinol.

[38] Harmon JM, Norman MR, Fowlkes BJ, Thompson EB (1979). Dexamethasone induces irreversible G1 arrest and death of a human lymphoid cell line. J Cell Physiol.

[39] He B, Zhang N, Zhao R (2016). Dexamethasone downregulates SLC7A5 expression and promotes cell cycle arrest, autophagy and apoptosis in BeWo cells. J Cell Physiol.

[40] Mattern J, Buchler MW, Herr I (2007). Cell cycle arrest by glucocorticoids may protect normal tissue and solid tumors from cancer therapy. Cancer Biol Ther.

[41] Yuan J, Adamski R, Chen J (2010). Focus on histone variant H2AX: to be or not to be. FEBS Lett.

[42] Rogakou EP, Pilch DR, Orr AH, Ivanova VS, Bonner WM (1998). DNA double-stranded breaks induce histone H2AX phosphorylation on serine 139. J Biol Chem.

[43] Mirza-Aghazadeh-Attari M, Mohammadzadeh A, Yousefi B, Mihanfar A, Karimian A, Majidinia M (2019). 53BP1: A key player of DNA damage response with critical functions in cancer. DNA Repair.

[44] Rappold I, Iwabuchi K, Date T, Chen J (2001). Tumor suppressor p53 binding protein 1 (53BP1) is involved in DNA damage-signaling pathways. J Cell Biol.

[45] Wieler S, Gagne JP, Vaziri H, Poirier GG, Benchimol S (2003). Poly(ADP-ribose) polymerase-1 is a positive regulator of the p53-mediated G1 arrest response following ionizing radiation. J Biol Chem.

[46] Hu Z, Holzschuh J, Driever W (2015). Loss of DDB1 leads to transcriptional p53 pathway activation in proliferating cells, cell cycle deregulation, and apoptosis in Zebrafish embryos. PLoS ONE.

[47] Hu J, McCall CM, Ohta T, Xiong Y (2004). Targeted ubiquitination of CDT1 by the DDB1-CUL4A-ROC1 ligase in response to DNA damage. Nat Cell Biol.

[48] Lovejoy CA, Lock K, Yenamandra A, Cortez D (2006). DDB1 maintains genome integrity through regulation of Cdt1. Mol Cell Biol.

[49] Senga T, Sivaprasad U, Zhu W, Park JH, Arias EE, Walter JC (2006). PCNA is a cofactor for Cdt1 degradation by CUL4/DDB1-mediated N-terminal ubiquitination. J Biol Chem.

[50] Knott GJ, Bond CS, Fox AH (2016). The DBHS proteins SFPQ, NONO and PSPC1: a multipurpose molecular scaffold. Nucleic Acids Res.

[51] de Silva HC, Lin MZ, Phillips L, Martin JL, Baxter RC (2019). IGFBP-3 interacts with NONO and SFPQ in PARP-dependent DNA damage repair in triple-negative breast cancer. Cell Mol Life Sci.

[52] Jaafar L, Li Z, Li S, Dynan WS (2017). SFPQ*NONO and XLF function separately and together to promote DNA double-strand break repair via canonical nonhomologous end joining. Nucleic Acids Res.

[53] Johnson TA, Chereji RV, Stavreva DA, Morris SA, Hager GL, Clark DJ (2018). Conventional and pioneer modes of glucocorticoid receptor interaction with enhancer chromatin in vivo. Nucleic Acids Res.

[54] Shen P, Cao X, Sun L, Qian Y, Wu B, Wang X (2021). KLF9 suppresses cell growth and induces apoptosis via the AR pathway in androgen-dependent prostate cancer cells. Biochem Biophys Rep.

[55] Sun J, Wang B, Liu Y, Zhang L, Ma A, Yang Z (2014). Transcription factor KLF9 suppresses the growth of hepatocellular carcinoma cells in vivo and positively regulates p53 expression. Cancer Lett.

[56] Du F, Sun L, Chu Y, Li T, Lei C, Wang X (2018). DDIT4 promotes gastric cancer proliferation and tumorigenesis through the p53 and MAPK pathways. Cancer Commun.

[57] Han N, Yuan F, Xian P, Liu N, Liu J, Zhang H (2019). GADD45a mediated cell cycle inhibition is regulated By P53 in bladder cancer. Onco Targets Ther.

[58] Sossey-Alaoui K, Pluskota E, Szpak D, Plow EF (2019). The Kindlin2-p53-SerpinB2 signaling axis is required for cellular senescence in breast cancer. Cell Death Dis.

[59] Zhang XM, Wang T, Hu P, Li B, Liu H, Cheng YF (2019). SERPINB2 overexpression inhibited cell proliferation, invasion and migration, led to G2/M arrest, and increased radiosensitivity in nasopharyngeal carcinoma cells. J Radiat Res.

[60] Matlashewski G, Banks L, Pim D, Crawford L (1986). Analysis of human p53 proteins and mRNA levels in normal and transformed cells. Eur J Biochem.

[61] Cuella-Martin R, Oliveira C, Lockstone HE, Snellenberg S, Grolmusova N, Chapman JR (2016). 53BP1 integrates DNA repair and p53-dependent cell fate decisions via distinct mechanisms. Mol Cell.

[62] Soussi T, Kroemer G (2017). TP53 and 53BP1 reunited. Trends Cell Biol.

[63] Flores-Alcantar A, Gonzalez-Sandoval A, Escalante-Alcalde D, Lomeli H (2011). Dynamics of expression of ARID1A and ARID1B subunits in mouse embryos and in cells during the cell cycle. Cell Tissue Res.

[64] Li H, Qian W, Weng X, Wu Z, Li H, Zhuang Q (2012). Glucocorticoid receptor and sequential P53 activation by dexamethasone mediates apoptosis and cell cycle arrest of osteoblastic MC3T3-E1 cells. PLoS One.

[65] Nagl NG, Patsialou A, Haines DS, Dallas PB, Beck GR, Moran E (2005). The p270 (ARID1A/SMARCF1) subunit of mammalian SWI/SNF-related complexes is essential for normal cell cycle arrest. Cancer Res.

[66] Prekovic S, Schuurman K, Mayayo-Peralta I, Manjon AG, Buijs M, Yavuz S (2021). Glucocorticoid receptor triggers a reversible drug-tolerant dormancy state with acquired therapeutic vulnerabilities in lung cancer. Nat Commun.

[67] Trotter KW, King HA, Archer TK (2015). Glucocorticoid receptor transcriptional activation via the BRG1-dependent recruitment of TOP2beta and Ku70/86. Mol Cell Biol.

[68] Pommier Y, Nussenzweig A, Takeda S, Austin C (2022). Human topoisomerases and their roles in genome stability and organization. Nat Rev Mol Cell Biol.

[69] Branzei D, Foiani M (2008). Regulation of DNA repair throughout the cell cycle. Nat Rev Mol Cell Biol.

[70] Ward IM, Minn K, Jorda KG, Chen J (2003). Accumulation of checkpoint protein 53BP1 at DNA breaks involves its binding to phosphorylated histone H2AX. J Biol Chem.

[71] Chowdhury D, Keogh MC, Ishii H, Peterson CL, Buratowski S, Lieberman J (2005). gamma-H2AX dephosphorylation by protein phosphatase 2A facilitates DNA double-strand break repair. Mol Cell.

[72] Sharma A, Singh K, Almasan A. Histone H2AX phosphorylation: a marker for DNA damage. Vol. 920. Totowa, NJ: Humana Press; 2012. 10.1007/978-1-61779-998-3_40 22941631

[73] Botuyan MV, Lee J, Ward IM, Kim JE, Thompson JR, Chen J (2006). Structural basis for the methylation state-specific recognition of histone H4-K20 by 53BP1 and Crb2 in DNA repair. Cell.

[74] Chapman JR, Sossick AJ, Boulton SJ, Jackson SP (2012). BRCA1-associated exclusion of 53BP1 from DNA damage sites underlies temporal control of DNA repair. J Cell Sci.

[75] Drane P, Brault ME, Cui G, Meghani K, Chaubey S, Detappe A (2017). TIRR regulates 53BP1 by masking its histone methyl-lysine binding function. Nature.

[76] Escribano-Diaz C, Orthwein A, Fradet-Turcotte A, Xing M, Young JT, Tkac J (2013). A cell cycle-dependent regulatory circuit composed of 53BP1-RIF1 and BRCA1-CtIP controls DNA repair pathway choice. Mol Cell.

[77] Kang Y, Cheong HM, Lee JH, Song PI, Lee KH, Kim SY (2011). Protein phosphatase 5 is necessary for ATR-mediated DNA repair. Biochem Biophys Res Commun.

[78] Wang B, Matsuoka S, Carpenter PB, Elledge SJ (2002). 53BP1, a mediator of the DNA damage checkpoint. Science.

[79] Krishnakumar R, Gamble MJ, Frizzell KM, Berrocal JG, Kininis M, Kraus WL (2008). Reciprocal binding of PARP-1 and histone H1 at promoters specifies transcriptional outcomes. Science.

[80] Wacker DA, Ruhl DD, Balagamwala EH, Hope KM, Zhang T, Kraus WL (2007). The DNA binding and catalytic domains of poly(ADP-ribose) polymerase 1 cooperate in the regulation of chromatin structure and transcription. Mol Cell Biol.

[81] Herceg Z, Wang ZQ (2001). Functions of poly(ADP-ribose) polymerase (PARP) in DNA repair, genomic integrity and cell death. Mutat Res.

[82] Sharma M, Zarnegar M, Li X, Lim B, Sun Z (2000). Androgen receptor interacts with a novel MYST protein, HBO1. J Biol Chem.

[83] Georgiakaki M, Chabbert-Buffet N, Dasen B, Meduri G, Wenk S, Rajhi L (2006). Ligand-controlled interaction of histone acetyltransferase binding to ORC-1 (HBO1) with the N-terminal transactivating domain of progesterone receptor induces steroid receptor coactivator 1-dependent coactivation of transcription. Mol Endocrinol.

[84] Niida H, Matsunuma R, Horiguchi R, Uchida C, Nakazawa Y, Motegi A (2017). Phosphorylated HBO1 at UV irradiated sites is essential for nucleotide excision repair. Nat Commun.

[85] Pardo M, Yu L, Shen S, Tate P, Bode D, Letney BL (2017). Myst2/Kat7 histone acetyltransferase interaction proteomics reveals tumour-suppressor Niam as a novel binding partner in embryonic stem cells. Sci Rep.

[86] Mittica G, Ghisoni E, Giannone G, Genta S, Aglietta M, Sapino A (2018). PARP inhibitors in ovarian cancer. Recent Pat Anticancer Drug Discov.

[87] MacPherson L, Anokye J, Yeung MM, Lam EYN, Chan YC, Weng CF (2020). HBO1 is required for the maintenance of leukaemia stem cells. Nature.

[88] Baell JB, Leaver DJ, Hermans SJ, Kelly GL, Brennan MS, Downer NL (2018). Inhibitors of histone acetyltransferases KAT6A/B induce senescence and arrest tumour growth. Nature.

[89] Kaelin WG (2005). The concept of synthetic lethality in the context of anticancer therapy. Nat Rev Cancer.

[90] Rajesh C, Baker DK, Pierce AJ, Pittman DL (2011). The splicing-factor related protein SFPQ/PSF interacts with RAD51D and is necessary for homology-directed repair and sister chromatid cohesion. Nucleic Acids Res.

[91] Visconti R, Della Monica R, Grieco D (2016). Cell cycle checkpoint in cancer: a therapeutically targetable double-edged sword. J Exp Clin Cancer Res.

[92] Buenrostro JD, Wu B, Chang HY, Greenleaf WJ. ATAC-seq: a method for assaying chromatin accessibility genome-wide. Curr Protoc Mol Biol. 2015;109:21 29 21–21 29 29. 10.1002/0471142727.mb2129s109 PMC4374986 25559105

[93] Conway-Campbell BL, George CL, Pooley JR, Knight DM, Norman MR, Hager GL (2011). The HSP90 molecular chaperone cycle regulates cyclical transcriptional dynamics of the glucocorticoid receptor and its coregulatory molecules CBP/p300 during ultradian ligand treatment. Mol Endocrinol.

[94] George CL, Birnie MT, Flynn BP, Kershaw YM, Lightman SL, Conway-Campbell BL (2017). Ultradian glucocorticoid exposure directs gene-dependent and tissue-specific mRNA expression patterns in vivo. Mol Cell Endocrinol.

[95] Love MI, Huber W, Anders S (2014). Moderated estimation of fold change and dispersion for RNA-seq data with DESeq2. Genome Biol.

[96] Zhang Y, Liu T, Meyer CA, Eeckhoute J, Johnson DS, Bernstein BE (2008). Model-based analysis of ChIP-Seq (MACS). Genome Biol.

[97] Heinz S, Benner C, Spann N, Bertolino E, Lin YC, Laslo P (2010). Simple combinations of lineage-determining transcription factors prime cis-regulatory elements required for macrophage and B cell identities. Mol Cell.

[98] Anders S, Huber W (2010). Differential expression analysis for sequence count data. Genome Biol.

[99] Trapnell C, Roberts A, Goff L, Pertea G, Kim D, Kelley DR (2012). Differential gene and transcript expression analysis of RNA-seq experiments with TopHat and Cufflinks. Nat Protoc.

