# Supplementary material for: Identification of a novel GR-ARID1a-P53BP1 protein complex involved in DNA damage repair and cell cycle regulation
Source: Oncogene. 2022 Nov 7;41(50):5347–60. doi: 10.1038/s41388-022-02516-2 (PMC9734058; doi:10.1038/s41388-022-02516-2)
Supplement: Supplementary file 1 — Supplementary figures [file 41388_2022_2516_MOESM1_ESM.docx]

**
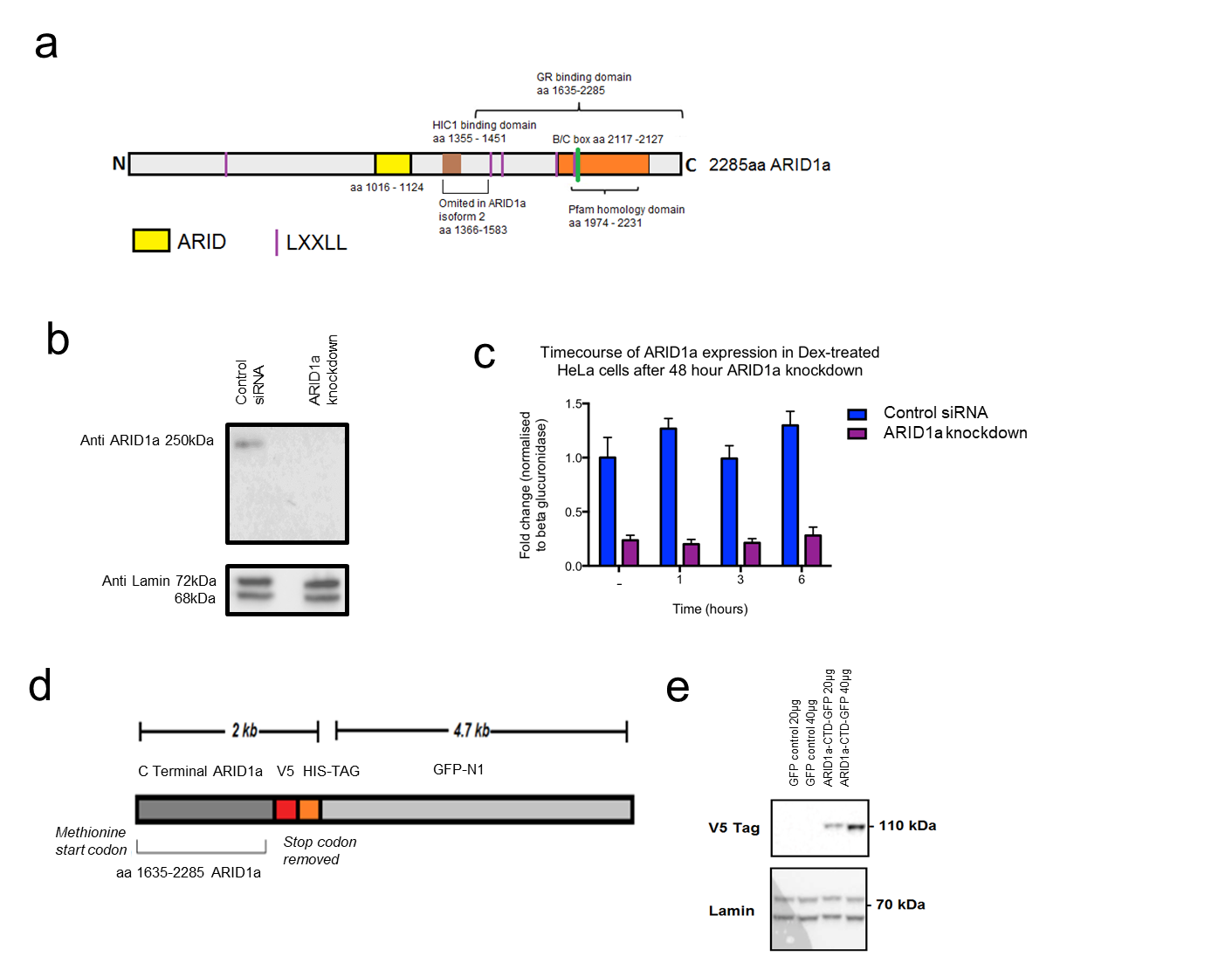
**

**
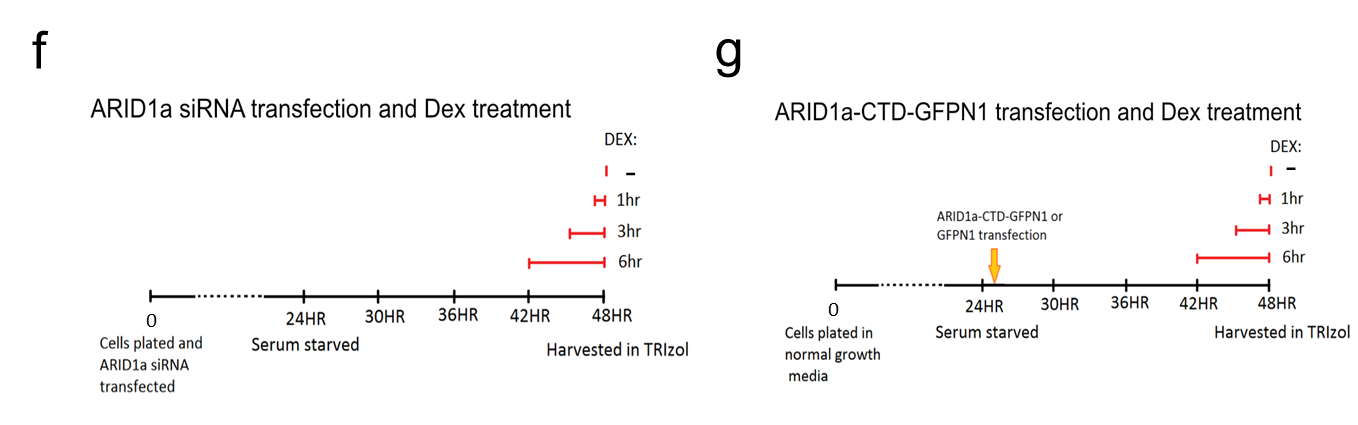
**

**Figure S1: ARID1a structure and siRNA knockdown of protein and mRNA. a)** ARID1a structure and GR binding region. **b)** Western blot showing ARID1a at 250kDa in scrambled control and no detectable protein following 48 hours reverse transfection with a combination of 4 ARID1a siRNAs. **c)** RT-qPCR reveals a reduction in ARID1a RNA expression following 48 hours ARID1a siRNA transfection over a 6 hour Dexamethasone timecourse. **d)** The ARID1a-CTD with a V5 and HIS-Tag within a GFPN1 construct (ARID1a-CTD-GFP). **e)** Western blot showing V5 tag of construct in transfected HeLa cell nuclear extracts compared to control cells. Lamin is used as an endogenous loading control. **f and g)** Schematic of transfection and Dex treatment of cells collected for RNA-Seq. **f)** Reverse transfection of ARID1a siRNA **g)** Transfection of ARID1a-CTD-GFPN1 construct following serum starvation.


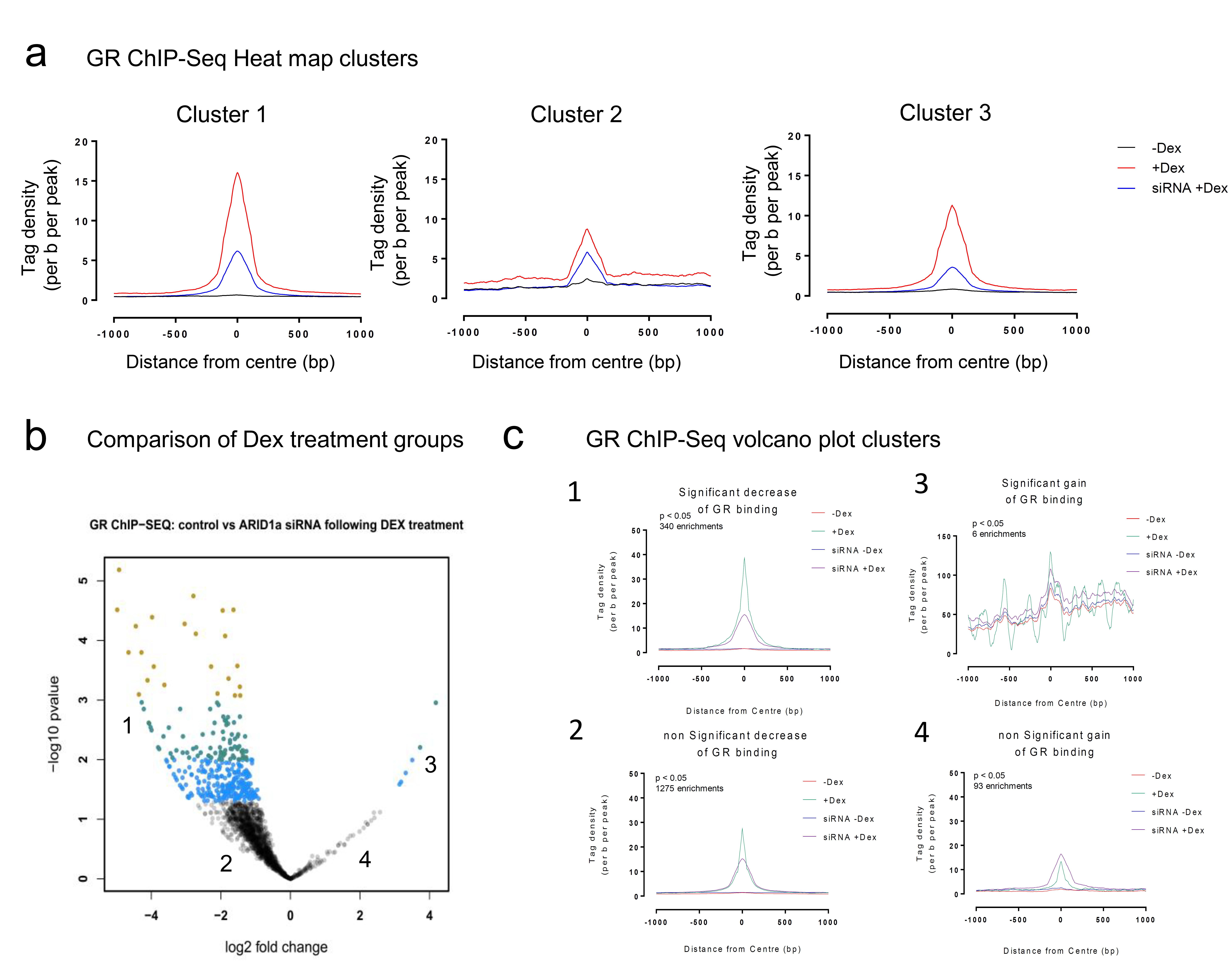


d


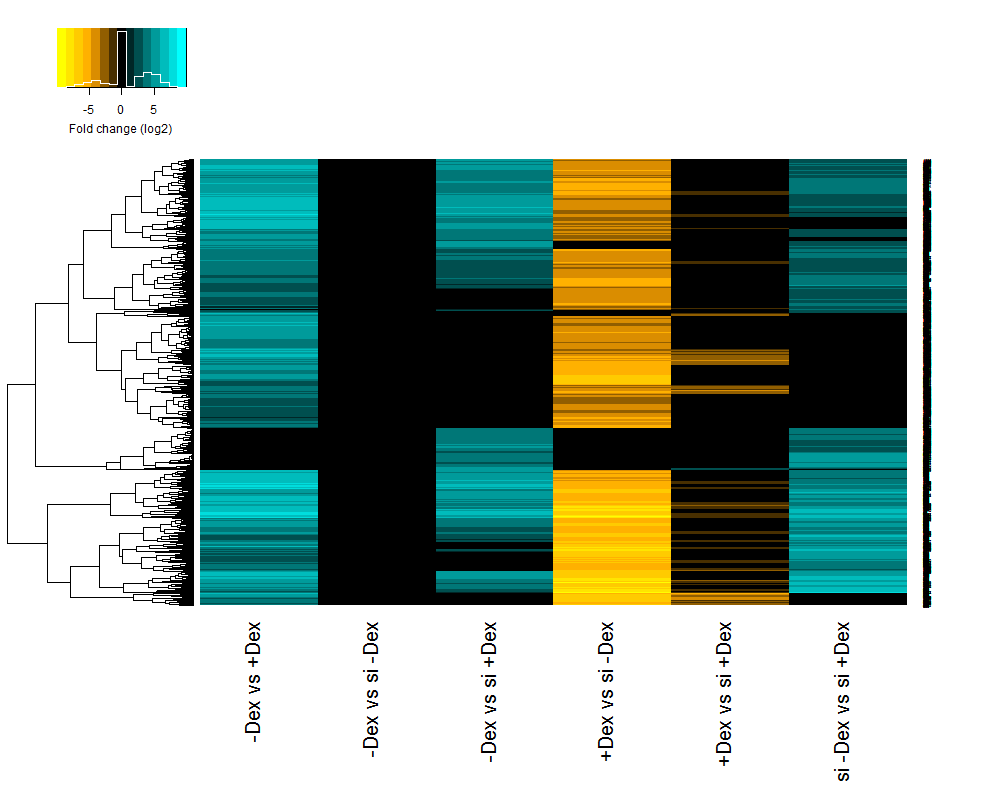

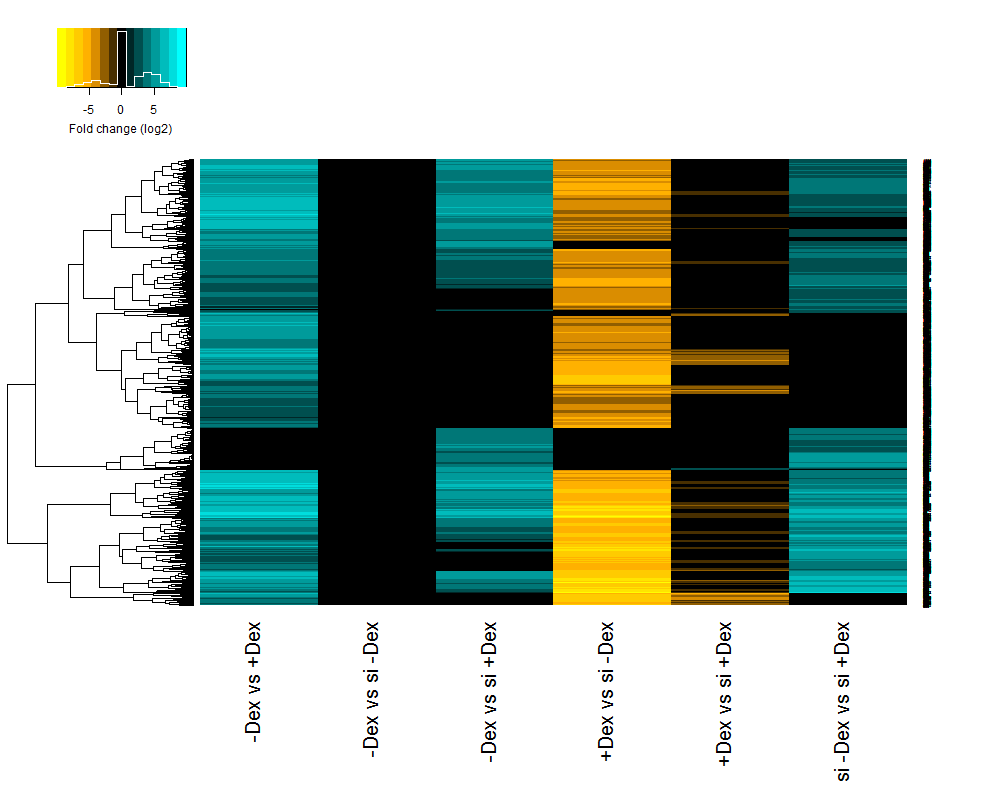

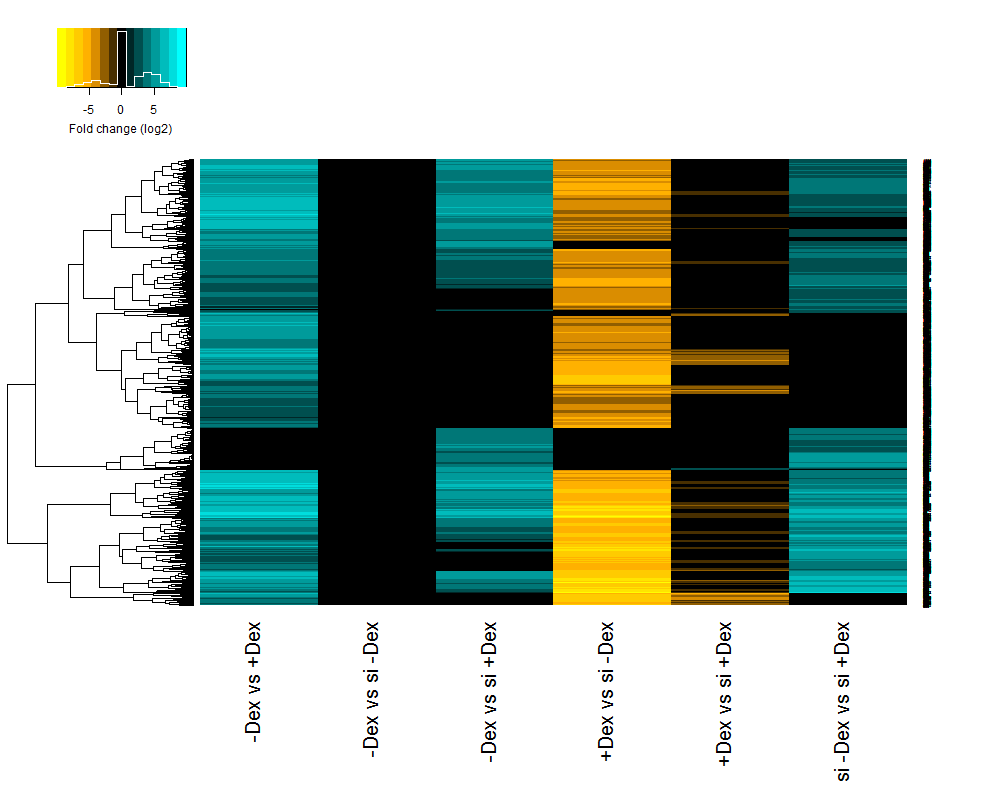


**Figure S2: GR ChIP-Seq data analysis; further analysis interrogating Figure 1b heatmap clusters, and comparisons between –Dex treatment conditions. a)** Histograms of GR peaks for 3 clusters identified in the GR ChIP-Seq heatmap. Heatmap Cluster 1 contained Dex induced GR binding sites where significant binding remained following ARID1a knockdown; supported here by the histogram. Heatmap Cluster 2 contained sites with an apparent gain in GR binding following ARID1a knockdown; however this is not supported here by the histogram which reveals a higher background for the control group and minimal difference in peak sizes. Heatmap Cluster 3 contained sites with a loss of significant GR binding; supported here by the histogram. **b)** Further analysis comprised a direct comparison of Dex treatment groups (+Dex control versus +Dex siRNA). Here, the data was visualized using a volcano plot. The volcano plot shows Log2 fold change of GR binding in (ARID1a + dex) relative to (control + dex) conditions (X axis), alongside –Log10 p value (Y axis). Significant changes in GR binding are indicated by blue (p < 0.05), green (p < 0.01), orange (p < 0.001). Following ARID1a siRNA knockdown volcano plot clusters 1-4 represent: 1) Significant decrease GR binding 2) Non-significant decrease GR binding 3) Significant gain GR binding 4) non-significant gain GR binding. **c)** Histograms for the 4 clusters determined by the volcano plot show changes in GR binding between control and siRNA in the absence and presence of Dex. Genes with a significant gain in GR binding appear to be a result of high background noise. The six gained regions in this comparison are the most robust gained sites from the comparison of -Dex control versus +Dex ARID1a siRNA. Therefore, we conclude that identified sites of increased GR binding after ARID1a knockdown can be disregarded due to high background in the GR ChIP-Seq data at these loci. **d)** Lack of differential GR binding in the absence of Dex between ARID1a knockdown and siRNA control HeLa cells. Heatmap of GR ChIP-SEQ data comparing control siRNA HeLa cells in the absence of Dex (-Dex) to control siRNA treated HeLa cells further treated with 100nM Dex for 30 min (+Dex), ARID1a siRNA knockdown –Dex (si –Dex) and ARID1a knockdown + Dex (si +Dex).

**Figure S3: Tag density heatmap and motif analysis of ATAC-Seq data.** **a)** ATAC-Seq tag density heatmap organised into the three predominant clusters of differentially open regions (DORs) identified in the z-score fold change heatmap (Figure 2). This shows that cluster I (containing groups 1, 2, 4 and 7) is comprised of sites with reduced pre-accessibility in ARID1a knockdown cells compared to siRNA control cells, cluster ii (containing groups 3, 5 and 6) is comprised of sites with increased pre-accessibility in ARID1a knockdown cells compared to siRNA control cells, and cluster iii (containing group 8 only) is comprised of sites that are Dex-inducible in both ARID1a knockdown and siRNA control cells. In this plot, there is improved visualisation of the reduced peak density in both -Dex and +Dex conditions in ARID1a knockdown compared to siRNA control cells, which explains why there is less difference seen in fold change of Dex-inducible chromatin remodelling at these sites. **b)** Motif analysis for the three clusters i, ii, iii. GREs and highly similar motifs are the most commonly detected motifs in cluster iii, but not clusters i and ii.

**Figure S4: SupraHex analysis of RNA-SEQ data and mRNA expression of common Dex inducible and repressible genes in HeLa cells with overexpression of the ARID1a-CTD compared to a control. a)** SupraHex analysis of high throughput RNA-SEQ data comparing ARID1a-CTD overexpression in HeLa cells to control cells following 100nM Dex treatment for - (no Dex control), 1, 3 and 6 hours. This represents a supra-hexagonal map in which genes are hierarchically clustered into smaller hexagons based on basal expression (calibrated to GFP -Dex) and the pattern of change in expression in response to Dex (Supplementary Figure 6 for further information). The Key indicates expression levels as log2 (FPKM data + 1); blue is low expression and red is high expression. **b,c,d,e)** RT-qPCR analysis to validate RNA-SEQ data. Two-way ANOVA shows a significant effect of Dex treatment time on **b)** Per1 expression (p < 0.0001), **c)** FKBP5 expression (p < 0.0001), **d)** PPP1R3C expression (p < 0.01), **e)** NR3C1 expression (p < 0.05). No significant effect of ARID1a-CTD-GFP overexpression b) Per1 (p = 0.3584, DFn = 1, DFd = 24), c) FKBP5 (p = 0.2242, DFn = 1, DFd = 40), d) PPP1R3C (p = 0.5609, DFn = 1, DFd = 15), e) NR3C1 (p = 0.3688, DFn = 1, DFd = 15). No significant interaction b) Per1 (p = 0.4557), c) FKBP5 (p = 0.5251), d) PPP1R3C (p = 0.0961) and e) NR3C1 (p = 0.3612). Bonferroni multiple comparisons tests reveal no significant difference between controls and ARID1a-CTD-GFP overexpression groups at any time point. Dunnett’s multiple comparisons tests confirm significant differences at each timepoint compared to the 0 hour control as indicated (* = p < 0.05, ** = p < 0.01, *** = p < 0.001, **** = p <0.0001).

**Figure S5: SupraHex analysis of high throughput RNA Sequencing data comparing ARID1a siRNA knock-down HeLa cells (represented by siRNA (Dex treatment time)) to control cells (represented by Control (Dex treatment time)) following 100nM Dex treatment for - (no Dex control), 1, 3 and 6 hours. a)** A supra-hexagonal map in which genes are hierarchically clustered into smaller hexagons as shown in **(b)** based on basal expression, the pattern of change in expression in response to Dex. Levels of basal expression are shown in the no treatment groups, Control - and SiRNA -. The Key indicates expression levels; blue is low expression and red is high expression. Induction or repression in response to Dex treatment in control cells (Control 1h, Control 3h, Control 6h) and knock-down cells (SiRNA 1h, SiRNA 3h, SiRNA 6h) is indicated by a shift in colour (e.g. induction represented by a shift from blue to red) in each smaller hexagon (node). **b)** The architectural design of each supraHex that contains 331 smaller hexagons known as nodes. **c)** The number of genes clustered into each node of the supraHex. Node numbering begins from the central node and then expands anticlockwise circularly outwards positioning data into the calibrator (Control -) supraHex based on basal expression but also taking into account the level of change in expression on Dex treatment as shown in image d. **d)** Nodes are positioned based on gene expression with basal expression from high (left), mid-expression (centre) to low (right). Nodes above the central horizontal axis show genes positioned based on induction pattern, with the most highly induced genes placed in the upper left corner of the supraHex. Nodes below the central horizontal axis show genes positioned based on a repression pattern, with the most highly repressible genes placed in the lower left corner of the supraHex. **e)** Nodes/genes are clustered again into 14 meta-clusters representing larger groups with similar expression and Dex responsive transcriptional profiles.

**Figure S6: SupraHex analysis of high throughput RNA-Seq data comparing ARID1a-CTD overexpression in HeLa cells (represented by ARID1a-CTD (Dex time interval)) to control cells (represented by GFP (Dex time interval)) following 100nM Dex treatment for - (no Dex control), 1, 3 and 6 hours. a)** A supra-hexagonal map in which genes are hierarchically clustered into smaller hexagons as shown in **(b)** based on basal expression and the pattern of change in expression in response to Dex. Levels of basal expression are shown in the no treatment groups, ARID1a-CTD- and GFP-. The Key indicates expression levels; blue is low expression and red is high expression. Induction or repression in response to Dex treatment in control cells (GFP1h, GFP3h, GFP6h) and C-terminal overexpression cells (ARID1a-CTD1h, ARID1a-CTD3h, ARID1a-CTD6h) is indicted by a shift in colour (e.g. induction represented by a shift from blue to red) in each smaller hexagon (node). **b)** The architectural design of each supraHex that contains 217 smaller hexagons known as nodes. **c)** The number of genes clustered into each node of the supraHex. Node numbering begins from the central node and then expands anticlockwise circularly outwards positioning data into the calibrator (GFP-) supraHex based on basal expression but also taking into account the level of change in expression on Dex treatment as shown in image d. **d)** Nodes are positioned based on gene expression with basal expression from high (right), mid-expression (centre) to low (left). Nodes below the central horizontal axis show genes positioned based on induction pattern, with highly induced genes placed in the lower half of the supraHex. Nodes above the central horizontal axis show genes positioned based on a repression pattern. **e)** Nodes/genes are clustered again into 10 meta-clusters representing larger groups with similar expression and Dex responsive transcriptional profiles.

**Figure S7: Increased γH2AX detected in HeLa cells expressing GFP-ARID1a-CTD (‘ARID1a-CTD-GFP’) versus GFP alone (‘GFP’) and EdU incorporation Flow cytometry analysis. a)** Increased γH2AX was associated with ARID1a-CTD expression at all timepoints assessed. The most pronounced effect was in serum starved cells, without DEX treatment. Levels of γH2AX reduced markedly at 12hr DEX treatment and increased again by 24hr DEX treatment albeit at significantly reduced levels when compared to cells without DEX treatment (-). Median and %CV is plotted on graph. Two-way ANOVA results are shown in table on RHS. There was a significant main effect of GFP-ARID1a-CTD expression, compared to GFP expression alone (P<0.0001), a significant main effect of DEX treatment time (P<0.0001) and a significant interaction (P<0.0001). Tukey’s multiple comparison post-test significant differences are shown on graph. **b)** Flow cytometry analysis with EdU incorporation over a Dex timecourse of ARID1a-CTD-GFP transfected cells compared to GFP controls. This shows a 2 hour EdU pulse chase following a Dex timecourse (-, 12 and 24 hours). This reveals a loss of proliferation in ARID1a-CTD-GFP overexpressing cells prior to and across the Dex timecourse compared to GFP controls. However, glucocorticoid mediated cell cycle arrest after 12 hours of Dex addition still occurs in both ARID1a-CTD-GFP overexpressing and GFP control groups. **c)** 24hr EdU incorporation in the absence (-) and presence (24hr) of Dex confirms a decrease in overall proliferation in ARID1a-CTD-GFP overexpressing cells compared to GFP. Also confirming cell cycle arrest in both groups with addition of Dex.

**Figure S8: Expression profile of PARP9, PARP14 and PARP12 genes in ARID1a-CTD-GFP overexpressing cells compared to GFP overexpressing control cells.** **a)** PARP12, **b)** PARP9 and **c)** PARP14 have higher expression at, 0 hr, 3 hr and 6 hr Dex treatment, with overexpression of ARID1a-CTD-GFPN1 compared to GFP only controls. Recovery of expression to levels similar to controls occurs following 1 hour of Dex treatment. Statistical significance of differential expression between the ARID1a-CTD overexpression and control group is calculated as log2 (FPKM value control vs FPKM value ARID1a-CTD overexpression). An adjusted P value is calculated and values < 0.05 after Benjamini-Hochberg correction for multiple testing are significant. * = p < 0.05, ** = p < 0.01. For differential gene expression statistical analysis see Trapnell et al., 2012.

**Figure S9: Chromatin associated GR co-immunoprecipitation data using Liquid chromatography mass spectrometry (LCMS).** LCMS results show loss of peptides (>80kDa) bound to GR (FDR 5%) resulting from ARID1a-CTD-GFP (‘ARID-CTD’) overexpression, including ARID1a, PARP1, P53BP1, DDB1, MSH6, KAT7, ATP-Dependent RNA Helicase A (DHX9). ARID1a-CTD-GFP was also detected to be bound to GR only in cells overexpressing the ARID1a-CTD-GFP.

**Supplementary Table S1: Functional pathway analysis showing changes in P53 pathway gene expression with ARID1a knockdown**

| **Functional pathway** | **Genes induced by 3 hours DEX treatment in the control condition only (i.e 0hr to 3hr).** | **Genes induced by 3 hours DEX treatment in the sirna knockdown group only.** | **Genes induced in both the control and sirna groups after 3 hours DEX** | **Genes repressed by 3 hours DEX treatment in the control condition only.** | **Genes repressed by 3 hours DEX treatment in the sirna knockdown group only.** | **Genes repressed in both the control and sirna groups after 3 hours DEX treatment** |
| --- | --- | --- | --- | --- | --- | --- |
| Genes involved in p53 pathways | ZNF365  SESN1  STEAP3  RGCC | BTG1  FOS  TSC22D1  KLF4  S100A10 | CDKN1A  GADD45A  JUN  SAT1  BTG2  FOXO3  NDRG1  DDIT4  SOCS1  TXNIP  STOM  SLC19A2  ITGB4  TSPYL2  SERPINB5  RALGDS  BCL2L1  DUSP1  CAV1  EDN2  MCL1  SERPINE1  PMAIP1  SNAI2  BTG2  TP53I3  DKK1  GADD45B  THBS1  TIMP3 | ATF3  SPHK1  PPP1R15A  RGS16  PDGFA  OSGIN1  CDKN2AIP | HSPA4L  SLC7A11  NUPR1  PPM1D  HIST3H2A  CCNG2  SESN2 | BMP2  LIF  TNFSF9  PLK2  IER5  HMOX1  TRAFD1 |
| Apoptosis |  |  | NFKBIA  PIK3R1  BIRC2  BIRC3  BCL2L1  IRAK3  PRKCD  GSN  DSP  PMAIP1  BMF  PRKCD  GSN  DSP  PMAIP1  BMF |  |  |  |
| Genes involved in Cell Cycle |  |  |  |  | H2AFX  HIST1H2AJ  HIST1H2AC  HIST1H2AB  HIST2H2AC  HIST1H2BL  HIST1H2BN  HIST2H2BE  HIST3H2BB | TGFB2  CDKN1B  CDKN2C |

**Table S2: Functional pathway analysis showing changes in P53 pathway gene expression with overexpression of ARID1a-CTD**

| **Functional pathway** | **Genes induced by 3 hours DEX treatment in the control condition only (i.e 0hr to 3hr).** | **Genes induced by 3 hours DEX treatment in the ARID1a-CTD O/X group only.** | **Genes induced in both the control and ARID1a-CTD O/X groups after 3 hours DEX** | **Genes repressed by 3 hours DEX treatment in the control condition only.** | **Genes repressed by 3 hours DEX treatment in the ARID1a-CTD O/X group only** | **Genes repressed in both the control and ARID1a-CTD O/X after 3 hours DEX treatment** |
| --- | --- | --- | --- | --- | --- | --- |
| Genes involved in p53 pathways |  | JUN  NDRG1  S100A10  ZFP36L1  BCL2L1 | CDKN1A  GADD45A  SAT1  BTG2  FOXO3  DDIT4  TXNIP  STOM  SLC19A2  ITGB4  TSPYL2  SERPINB5  DUSP1  CAV1  EDN2  MCL1  SERPINE1  SNAI2  RGCC  GADD45B  THBS1 |  |  | BMP2  PLK2  RGS16  PDGFA |
| Apoptosis |  | JUN  BCL2L1  WEE1 | NFKBIA  PIK3R1  BIRC3  IRAK3  CDKN1A  GADD45A  MCL1  GADD45B  SAT1  BTG2  CAV1  IFNGR1  EMP1  TXNIP  BMF |  |  | BMP2  IL6  TNFRSF12A |
| Genes involved in Cell Cycle |  |  | GADD45A  GADD45B  CDKN1A  CDKN1C |  |  |  |

**Table S3: Histone genes with altered expression levels with the overexpression of the ARID1a-CTD**

| **Lower basal expression in ARID1a-CTD overexpression cells vs control (0hr vs 0hr)** | **No change in expression levels following 1 hour Dex in ARID1a-CTD overexpression cells vs controls (1hr vs 1hr)** | **Significant induction in ARID1a-CTD overexpression cells only following 1 hour Dex (0hr vs 1hr)** | **Lower expression in**  **ARID1a-CTD overexpression cells following 3 hours Dex (3hr vs 3hr)** | **Repressed in control cells only following 6 hours of Dex (0hr vs 6hr).** | **Lower expression in ARID1a-CTD overexpression cells vs control following 6 hours Dex (6hr vs 6hr)** |
| --- | --- | --- | --- | --- | --- |
| HIST1H2AM  HIST1H4E  HIST1H2BG  HIST1H3B  HIST1H3H  HIST1H2BH  HIST1H3G  HIST1H2AK  HIST1H2AJ  HIST1H2BC  HIST1H4H  HIST1H1C  HIST1H2AE  HIST1H1D  HIST1H2BI  HIST1H1B  HIST1H3F  HIST1H2AG  HIST1H1A  HIST1H2AC  HIST1H2AB  HIST2H2AB  HIST1H3A  HIST1H4C  HIST1H2BD  HIST1H4D  HIST1H2AH  HIST2H3D  HIST1H2AI  HIST1H4B  H2AZ | HIST1H2AM  HIST1H4E  HIST1H2BG  HIST1H3B  HIST1H3H  HIST1H2BH  HIST1H3G  HIST1H2AK  HIST1H2AJ  HIST1H2BC  HIST1H4H  HIST1H1C  HIST1H2AE  HIST1H1D  HIST1H2BI  HIST1H1B  HIST1H3F  HIST1H2AG  HIST1H1A  HIST1H2AC  HIST1H2AB  HIST2H2AB  HIST1H3A  HIST1H4C  HIST1H2BD  HIST1H4D  HIST1H2AH  HIST2H3D  HIST1H2AI  HIST1H4B  H2AZ | HIST2H3D  HIST1H2AB  HIST3H2A  HIST2H2AB  HIST1H4B  HIST1H3H  HIST1H2AI  HIST1H2AK  HIST1H2AH  HIST1H3A  HIST1H3B  HIST1H2AC  HIST1H2AJ  HIST1H2AG  HIST1H4C  HIST1H3F  HIST1H2AL  HIST1H2BD | HIST1H2AM  HIST1H4E  HIST1H2BG  HIST1H3B  HIST1H3H  HIST1H2BH  HIST1H3G  HIST1H2AK  HIST1H2AJ  HIST1H2BC  HIST1H4H  HIST1H1C  HIST1H2AE  HIST1H1D  HIST1H2BI  HIST1H1B  HIST1H3F  HIST1H2AG  HIST1H1A  HIST1H2AC  HIST1H2AB  HIST2H2AB  HIST1H3A  HIST1H4C  HIST1H2BD  HIST1H4D  HIST1H2AH  HIST2H3D  HIST1H2AI  HIST1H4B  HIST2H2AC  HIST1H2BO  HIST1H1E  HIST1H2BN  HIST1H2BF  HIST1H4A  HIST1H2AL  HIST2H2BF  HIST1H2BL  HIST3H2A  HIST1H3D | HIST1H2BD  HIST1H2BC  HIST1H1B  HIST1H4C  HIST1H3F  HIST1H2AH  HIST1H2AE  HIST1H3G  HIST1H3C  HIST1H2AI  HIST1H2AK  HIST1H2BG  HIST1H2BL  HIST3H2BB  HIST1H4D  HIST1H3A  HIST1H2BI  HIST1H4B  HIST1H2BM | HIST1H2BD  HIST1H2AI  HIST1H4B |

**Table S4: LCMS data showing 61 proteins that do not interact (fold change < 2 at FDR <0.01) with GR when ARID1a is knocked down compared to controls**

* Represents the proteins that are involved in the DNA damage or repair response (DAVID Bioinformatics resources version 6.8).

| Protein accession number | Description |
| --- | --- |
| O75643 | U5 small nuclear ribonucleoprotein 200 kDa helicase OS=Homo sapiens GN=SNRNP200 PE=1 SV=2 - [U520_HUMAN] |
| P78527* | DNA-dependent protein kinase catalytic subunit OS=Homo sapiens GN=PRKDC PE=1 SV=3 - [PRKDC_HUMAN] |
| P42166 | Lamina-associated polypeptide 2, isoform alpha OS=Homo sapiens GN=TMPO PE=1 SV=2 - [LAP2A_HUMAN] |
| Q9Y487 | V-type proton ATPase 116 kDa subunit a isoform 2 OS=Homo sapiens GN=ATP6V0A2 PE=1 SV=2 - [VPP2_HUMAN] |
| Q16531* | DNA damage-binding protein 1 OS=Homo sapiens GN=DDB1 PE=1 SV=1 - [DDB1_HUMAN] |
| Q08945 | FACT complex subunit SSRP1 OS=Homo sapiens GN=SSRP1 PE=1 SV=1 - [SSRP1_HUMAN] |
| A0A024R1Y2 | ATP-citrate synthase OS=Homo sapiens GN=ACLY PE=3 SV=1 - [A0A024R1Y2_HUMAN] |
| P19338 | Nucleolin OS=Homo sapiens GN=NCL PE=1 SV=3 - [NUCL_HUMAN] |
| B4DVQ0 | cDNA FLJ58286, highly similar to Actin, cytoplasmic 2 OS=Homo sapiens PE=2 SV=1 - [B4DVQ0_HUMAN] |
| O60568 | Procollagen-lysine,2-oxoglutarate 5-dioxygenase 3 OS=Homo sapiens GN=PLOD3 PE=1 SV=1 - [PLOD3_HUMAN] |
| B4DHG3 | cDNA FLJ55329, highly similar to Procollagen-lysine,2-oxoglutarate5-dioxygenase 2 (EC 1.14.11.4) OS=Homo sapiens PE=2 SV=1 - [B4DHG3_HUMAN] |
| Q9BSV4* | SFPQ protein (Fragment) OS=Homo sapiens GN=SFPQ PE=2 SV=2 - [Q9BSV4_HUMAN] |
| Q13619* | Cullin-4A OS=Homo sapiens GN=CUL4A PE=1 SV=3 - [CUL4A_HUMAN] |
| Q8TCS8 | Polyribonucleotide nucleotidyltransferase 1, mitochondrial OS=Homo sapiens GN=PNPT1 PE=1 SV=2 - [PNPT1_HUMAN] |
| B3KXS7 | cDNA FLJ45975 fis, clone PLACE7018479, highly similar to 182 kDa tankyrase 1-binding protein OS=Homo sapiens PE=2 SV=1 - [B3KXS7_HUMAN] |
| P01024 | Complement C3 OS=Homo sapiens GN=C3 PE=1 SV=2 - [CO3_HUMAN] |
| Q9UIG0* | Tyrosine-protein kinase BAZ1B OS=Homo sapiens GN=BAZ1B PE=1 SV=2 - [BAZ1B_HUMAN] |
| O95782 | AP-2 complex subunit alpha-1 OS=Homo sapiens GN=AP2A1 PE=1 SV=3 - [AP2A1_HUMAN] |
| I3L2C7 | Gem-associated protein 4 OS=Homo sapiens GN=GEMIN4 PE=1 SV=1 - [I3L2C7_HUMAN] |
| E5KLK2 | Mitochondrial dynamin-like 120 kDa protein OS=Homo sapiens GN=OPA1 PE=4 SV=1 - [E5KLK2_HUMAN] |
| C9J8U1 | Cytospin-A (Fragment) OS=Homo sapiens GN=SPECC1L PE=4 SV=7 - [C9J8U1_HUMAN] |
| O95251 | Histone acetyltransferase KAT7 OS=Homo sapiens GN=KAT7 PE=1 SV=1 - [KAT7_HUMAN] |
| A0A087WWJ1* | DNA mismatch repair protein Msh6 OS=Homo sapiens GN=MSH6 PE=1 SV=1 - [A0A087WWJ1_HUMAN] |
| Q02539 | Histone H1.1 OS=Homo sapiens GN=HIST1H1A PE=1 SV=3 - [H11_HUMAN] |
| Q8NBJ5 | Procollagen galactosyltransferase 1 OS=Homo sapiens GN=COLGALT1 PE=1 SV=1 - [GT251_HUMAN] |
| B4DVV1 | cDNA FLJ51526, highly similar to Homo sapiens aldehyde dehydrogenase 16 family, member A1 (ALDH16A1), mRNA OS=Homo sapiens PE=2 SV=1 - [B4DVV1_HUMAN] |
| E1NZA1 | Peroxisome proliferator activated receptor interacting complex protein OS=Homo sapiens GN=PRIC295 PE=2 SV=1 - [E1NZA1_HUMAN] |
| A0A125QYY5 | GCT-A9 light chain variable region (Fragment) OS=Homo sapiens PE=2 SV=1 - [A0A125QYY5_HUMAN] |
| P09874* | Poly [ADP-ribose] polymerase 1 OS=Homo sapiens GN=PARP1 PE=1 SV=4 - [PARP1_HUMAN] |
| B4DP93 | cDNA FLJ53437, highly similar to Major vault protein OS=Homo sapiens PE=2 SV=1 - [B4DP93_HUMAN] |
| Q7Z5Y0 | EIF4B protein (Fragment) OS=Homo sapiens GN=EIF4B PE=2 SV=1 - [Q7Z5Y0_HUMAN] |
| A0A087WZZ5 | Splicing factor 3B subunit 2 OS=Homo sapiens GN=SF3B2 PE=1 SV=1 - [A0A087WZZ5_HUMAN] |
| A0A024R872 | Chromosome 9 open reading frame 88, isoform CRA_a OS=Homo sapiens GN=C9orf88 PE=4 SV=1 - [A0A024R872_HUMAN] |
| B3KNE7 | cDNA FLJ14468 fis, clone MAMMA1000734, highly similar to Translocation protein SEC63 homolog OS=Homo sapiens PE=2 SV=1 - [B3KNE7_HUMAN] |
| Q9Y597 | BTB/POZ domain-containing protein KCTD3 OS=Homo sapiens GN=KCTD3 PE=1 SV=2 - [KCTD3_HUMAN] |
| B4E2Z3 | cDNA FLJ54090, highly similar to 4F2 cell-surface antigen heavy chain OS=Homo sapiens PE=2 SV=1 - [B4E2Z3_HUMAN] |
| Q6NSB3 | Alpha-amylase (Fragment) OS=Homo sapiens GN=AMY1A PE=2 SV=1 - [Q6NSB3_HUMAN] |
| Q05BS0 | Eukaryotic translation initiation factor 3 subunit A (Fragment) OS=Homo sapiens GN=EIF3A PE=2 SV=1 - [Q05BS0_HUMAN] |
| B7Z3V1 | Sodium/potassium-transporting ATPase subunit alpha (Fragment) OS=Homo sapiens PE=2 SV=1 - [B7Z3V1_HUMAN] |
| Q9H8E3 | cDNA FLJ13715 fis, clone PLACE2000404, moderately similar to PROBABLE LEUCYL-TRNA SYNTHETASE (EC 6.1.1.4) OS=Homo sapiens PE=2 SV=1 - [Q9H8E3_HUMAN] |
| B4DZH7 | cDNA FLJ56367 OS=Homo sapiens PE=2 SV=1 - [B4DZH7_HUMAN] |
| Q8IVJ7 | AP20 region protein OS=Homo sapiens GN=APRG1 PE=2 SV=1 - [Q8IVJ7_HUMAN] |
| B4DTT8 | cDNA FLJ61396 OS=Homo sapiens PE=2 SV=1 - [B4DTT8_HUMAN] |
| B7Z5C1 | cDNA FLJ56126, highly similar to Programmed cell death 6-interacting protein OS=Homo sapiens PE=2 SV=1 - [B7Z5C1_HUMAN] |
| H3BPK7 | Alanine--tRNA ligase, cytoplasmic (Fragment) OS=Homo sapiens GN=AARS PE=1 SV=3 - [H3BPK7_HUMAN] |
| K7EMH3 | DNA-directed RNA polymerase, mitochondrial (Fragment) OS=Homo sapiens GN=POLRMT PE=1 SV=4 - [K7EMH3_HUMAN] |
| B3KRR1 | cDNA FLJ34725 fis, clone MESAN2005958, highly similar to RNA-binding protein Luc7-like 2 OS=Homo sapiens PE=2 SV=1 - [B3KRR1_HUMAN] |
| Q15758 | Neutral amino acid transporter B(0) OS=Homo sapiens GN=SLC1A5 PE=1 SV=2 - [AAAT_HUMAN] |
| B4DSD0 | cDNA FLJ59409, highly similar to Peroxisomal multifunctional enzyme type 2 OS=Homo sapiens PE=2 SV=1 - [B4DSD0_HUMAN] |
| B7Z602 | cDNA FLJ59408, highly similar to 150 kDa oxygen-regulated protein (Orp150) OS=Homo sapiens PE=2 SV=1 - [B7Z602_HUMAN] |
| I3L2J8 | Centrosomal protein of 131 kDa OS=Homo sapiens GN=CEP131 PE=1 SV=1 - [I3L2J8_HUMAN] |
| Q6LAM1 | Heavy chain of factor I (Fragment) OS=Homo sapiens PE=2 SV=1 - [Q6LAM1_HUMAN] |
| Q6DEN2 | DPYSL3 protein OS=Homo sapiens GN=DPYSL3 PE=2 SV=1 - [Q6DEN2_HUMAN] |
| B3KQG6 | cDNA FLJ90427 fis, clone NT2RP3000481, highly similar to Importin-7 OS=Homo sapiens PE=2 SV=1 - [B3KQG6_HUMAN] |
| H0Y488 | AT-rich interactive domain-containing protein 1A (Fragment) OS=Homo sapiens GN=ARID1A PE=1 SV=1 - [H0Y488_HUMAN] |
| M0R142* | Tumor suppressor p53-binding protein 1 (Fragment) OS=Homo sapiens GN=TP53BP1 PE=1 SV=1 - [M0R142_HUMAN] |
| A0PJ71* | NBN protein (Fragment) OS=Homo sapiens GN=NBN PE=2 SV=1 - [A0PJ71_HUMAN] |
| B4DY72 | cDNA FLJ52360, highly similar to Heat-shock protein 105 kDa OS=Homo sapiens PE=2 SV=1 - [B4DY72_HUMAN] |
| B7Z502 | cDNA FLJ61327, highly similar to Exocyst complex component 4 OS=Homo sapiens PE=2 SV=1 - [B7Z502_HUMAN] |
| B3KNR6 | cDNA FLJ30255 fis, clone BRACE2002444, highly similar to LAS1-like protein OS=Homo sapiens PE=2 SV=1 - [B3KNR6_HUMAN] |
| A8K9K4 | cDNA FLJ75504, highly similar to Homo sapiens glucosidase I, mRNA OS=Homo sapiens PE=2 SV=1 - [A8K9K4_HUMAN] |

**Table S5: SiRNA ARID1a sequences (Dharmacon, UK)**

| **siRNA** | **Target Sequence** |
| --- | --- |
| ARID1a siRNA 1 | GAAUAGGGCCUGAGGGAAA |
| ARID1a siRNA 2 | AGAUGUGGGUGGACCGUUA |
| ARID1a siRNA 3 | GCAACGACAUGAUUCCUAU |
| ARID1a siRNA 4 | GGACCUCUAUCGCCUCUAU |
| ON-TARGET plus Non-targeting pool | UGGUUUACAUGUCGACUAA,  UGGUUUACAUGUUGUGUGA,  UGGUUUACAUGUUUUCUGA,  UGGUUUACAUGUUUUCCUA |

**Table S6: ChIP primers GR and Pol2**

| **Human gene of interest:** | **Forward primer sequence:**  **5’ – 3’** | **Reverse primer sequence:**  **5’ – 3’** |
| --- | --- | --- |
| Per1 Distal GRE | ACAGGACGGCTGTCGTTTTG | CGCACTTGGGAACATCATGT |
| Per1 Proximal GRE (So et al., 2009) [1] | CTAGTCCGAAGTGGGCTGAC | CCGGTCTTCTTGCTCGTTAC |
| FKBP5 intron 1 GRE | CAGCCAGACCAAACAACTCC | CAGAGCAGAAGGTTCAGGGA |
| DUSP1 GRE | CTCCTCCTCCCTTTCCTTGG | TGGCCCCACATTGAAAATGG |
| Per1 Exon 19 | GCCTTGGTGCTCCCTAACTA | TCTGGAGTGCCCCATAAGGA |
| FKBP5 intron 5 | AATAAGAAAACACGGGCCGG | TCTCGGCTCACTACAACCTC |
| TSC22D1 intron 1 | GTCTTGCTGTGTCACCCATG | TGGGGTCAGGAGTTCAAGAC |
| KLF4 exon 2 | ACCTGGCGAGTCTGACATG | CACACCCACGAAAACCCAC |

**[1]** So AY, Bernal TU, Pillsbury ML, Yamamoto KR, Feldman BJ. Glucocorticoid regulation of the circadian clock modulates glucose homeostasis. *Proc Natl Acad Sci U S A* 2009; 106: 17582-17587.

**Table S7: mRNA Primer sequences**

| **Human Gene of interest:** | **Forward primer sequence:**  **5’ – 3’** | **Reverse primer sequence:**  **5’ – 3’** |
| --- | --- | --- |
| β glucuronidase | CTAACTATGCAGCAGACAAGG | GATACCAAGAGTAGTAGCTGTTC |
| Period 1 | CACTGGCCTGTGTCAAGC | GTGTACTCAGACGTGATGTG |
| ARID1A | TATATGCAGAGGAACCCCCAGAT | CATTGGGCAAGGCATTATAGTT |
| ARID1B | CTCAGCACAAAGCAGGCAAG | AGGGAGAGCTGGAAGCCATA |
| FKBP5 | CAAAGGCCAAGTCATCAAGG | TTCGAGGGAATTTTAGGGAGA |
| DUSP1 | CCTGACAGCGCGGAATCT | GATTTCCACCGGGCCAC |
| H2AX | ACGAGGAGCTCAACAAGCTG | CGGGCCCTCTTAGTACTCCT |
| STEAP3 | GGCCTTCAATGTCATCTCTGC | GCTTGGCTTCTGGCTGGTC |
| TSC22D1 | TCAGGACCAACCGCAAGCT | CTTCTCCGTCTGTTCAGTTCACA |
| KLF4 | CGAACCCACACAGGTGAGAA | TACGGTAGTGCCTGGTCAGTTC |
| PPP1R3C | GCGTTGTGTTTGCTGACTCC | CGGTTGAAGGCTGAGGGAAAT |
| NR3C1 | AGTGATTGCAGCAGTGAATG | TGCAGTAGGGTCATTTGGTCAT |
| PARP14 | AGACACAAAGGGCCACAG | CCAGAGATCTGGATTCTG |
